# Polymorphism of the Oxytocin Receptor Gene Modulates Behavioral and Attitudinal Trust among Men but Not Women

**DOI:** 10.1371/journal.pone.0137089

**Published:** 2015-10-07

**Authors:** Kuniyuki Nishina, Haruto Takagishi, Miho Inoue-Murayama, Hidehiko Takahashi, Toshio Yamagishi

**Affiliations:** 1 Graduate School of Brain Sciences, Tamagawa University, Tokyo, Japan; 2 Brain Science Institute, Tamagawa University, Tokyo, Japan; 3 Wildlife Research Center, Kyoto University, Kyoto, Japan; 4 Department of Psychiatry, Graduate School of Medicine, Kyoto University, Kyoto, Japan; 5 Graduate School of International Corporate Strategy, Hitotsubashi University, Tokyo, Japan; University of Tokyo, JAPAN

## Abstract

A relationship between the oxytocin receptor gene (*OXTR*) and behavioral and attitudinal trust has been suggested, but the nature of this relationship has not yet been established. We obtained behavioral trust data from 470 Japanese participants (242 women) aged 20–59 years, together with their levels of general trust and personality traits (NEO-FFI). Saliva buccal swabs were collected from 411 of these 470 participants and used for genotyping of *OXTR* rs53576. Our participants were found to have more AA alleles (40%) than GG alleles (12%). The GG men were more trusting and also rated higher on attitudinal trust than AA men, and this difference did not diminish when personality traits were controlled for. However, this pattern was not observed among women. In addition, controlling for attitudinal trust reduced the difference in behavioral trust among men to a non-significant level, but the difference in attitudinal trust remained significant when behavioral trust was controlled. These results indicate that the *OXTR* genotype affects attitudinal trust as part of an individual’s relatively stable disposition, and further affects behavioral trust through changes in attitudinal trust.

## Introduction

Trust plays an integral role in effective interpersonal, social, economic, legal, and political functioning [1–5]. Although most trust-related research has been conducted by social scientists [1–5] who are predominantly concerned with social and psychological antecedents and consequences of trust, the past decade has witnessed a growing interest in the biological foundations of trust. One focus of this newly developing research is the role that oxytocin plays in modulating trust-related behavior [6–10].

Oxytocin is a nine-amino acid peptide produced in the paraventricular and supraoptic nuclei of the hypothalamus and secreted from the posterior pituitary glands. Traditionally, oxytocin has been known to play a role in social attachment [11–14]. More recently, the role that it plays in modulating human social behavior, particularly in reducing stress responses and promoting trust in interpersonal contexts, has been investigated. For example, plasma oxytocin levels were positively related to trusting behavior in a trust game [10]. Furthermore, intranasal administration of oxytocin has been found to enhance trust choices in trust games [6,7,9]. An fMRI study revealed that the increase in trust choices in trust game players receiving intranasal administration of oxytocin was accompanied by depressed activity in the amygdala [15]. The positive effect of oxytocin was limited to the social context, and it did not promote non-social risk propensity. Therefore, oxytocin is suggested to promote trusting behavior by reducing fear of social risks.

Another line of research regarding the biological foundations of trust comes from a finding in a twin study [16] that showed a high level of heritability of trusting behavior. Given this high heritability of trusting behavior, Krueger and colleagues [8] investigated genetic foundations of trust in the oxytocin receptor gene (*OXTR*), and found a relationship between rs53576 alleles and trusting behavior. Specifically, they found that individuals with the GG allele of rs53576 were more likely to trust in the trust game than those with either the AG or the AA allele. The oxytocin receptor (*OXTR*) gene is localized to human chromosome 3p25 and contains 4 exons and 3 introns [17]. rs53576, a single nucleotide polymorphism located in the third intron, is associated with socio-emotional phenotypes, including trusting behavior [8], empathy [18], emotional support seeking [19], pro-social disposition [20], self-punishment [21], stress reactivity [22], maternal sensitivity [23], and reward dependence [24].

The relationship between the GG allele of *OXTR* rs53576 and trusting behavior has not yet been fully established, however. At least one study found no relationship between the two [25]. Krueger at al. [8] attributed this inconsistency between their findings and those of Apicella et al. [25] to two possible modulators. The first was the use of a strategy method by Apicell et al. [25]. Using a strategy method, participants indicated how they would react to a set of situations in which their partners had transferred a particular portion of their endowments. Compared to the actual situation in which participants faced an actual transfer by their partners as in the study of Krueger et al. [8], participants who responded to the strategy method may have been less emotionally loaded. Krueger et al. thus suggested that the lack of strong emotional responses with the strategy method could explain the negative finding of Apicella et al. [25]. Another possible source of the negative findings of Apicell et al. was the difference in the sex composition of the participants. The results of Krueger et al. [8] are based on male-male pairs, whereas Apicella et al. [25] included both male and female participants.

The first goal of our study was to determine whether the positive relationship between the GG allele and trusting behavior in the trust game could be robustly observed across sexes even with the use of the strategy method. To achieve this goal, we included approximately equal numbers of men (N = 228) and women (N = 242) in our sample. Furthermore, ours is the first study to test the relationship between rs53576 and trusting behavior outside of Western populations, and thus a positive result would demonstrate the robustness of this relationship across cultures.

The second goal of our study was to examine whether the relationship of the GG allele with trust extended beyond the actual trusting behavior to more generalized attitudinal trust. This issue of how far the relationship extends has implications in two contexts. First, general attitudinal trust is less variable over time and different study designs, which leads us to expect that rs53576 alleles are more strongly related to attitudinal trust than to trusting behavior. Estimates of heritability of attitudinal trust in twin studies suggest that general attitudinal trust is partly heritable (33% for men and 39% for women [26], 14–31% [27], and 31% [28]), although one study suggests that the heritability of attitudinal trust (approximately 5%) is lower than that of trusting behavior (10–20%) [29]. Our results regarding the *OXTR*-trust relationship would thus provide a different perspective for the issue of heritability of attitudinal and behavioral trust.

General attitudinal trust is a fragile form of trust in the sense that it is quickly replaced by more specific trust [4]. However, it provides a foundation for the development of social capital, especially the bridging type that connects people across different groups and sectors of society [3]. People with high general trust give potential interaction partners the benefit of the doubt, that is, an assumption of trustworthiness until proven otherwise. Low initial levels of general trust lead individuals to avoid socially risky, yet potentially rewarding, relationships. The spread of individuals with low general trust who keep themselves away from potentially rewarding social relationships will lead a society to fragmentation into cohesive cliques. Given the importance of general attitudinal trust and the large cross-cultural differences in the level of attitudinal trust [4], in addition to the recent findings reporting large cultural differences in the distribution of rs53576 [19,21,30] roughly paralleling cultural differences in general attitudinal trust, demonstration of the genetic differences in general attitudinal trust would open a new field of research on gene-cultural co-evolution of human cooperation.

## Materials and Methods

### Participants

Six hundred non-student residents living in a relatively wealthy Tokyo suburb were selected from a list of 1,670 applicants who responded to a brochure that was distributed to about 180,000 households. These 600 individuals consisted of 75 men and 75 women in each 10-year age group from 20 to 59 years. Other demographic characteristics of this sample are presented in S1, S2, S3, S4 and S5 Figs. Of the 600 individuals, 564 participated in the initial wave of the study, in which demographic data were collected, along with information concerning participants’ personalities and social attitudes. The study was conducted in seven waves over a period of 32 months. Each wave lasted 3 to 6 hours, during which various economic games and cognitive experiments were conducted, and psychological measures were administered. Findings involving some of the variables collected in the seven waves, including the trust game and the measures of general attitudinal trust reported in this paper, have been reported elsewhere [31], but this is the first report of the relationship between *OXTR* and behavioral and attitudinal trust.

All experimental protocols were approved by the Ethics Committee of the Brain Science Institute, Tamagawa University, where the study was conducted, and by the ethics committee of Kyoto University Graduate School and Faculty of Medicine where the genotyping analysis was conducted. An informed consent form was signed by each participant. All tasks, including the trust game and responses to questionnaires, were performed in individual isolation compartments. Participants were not allowed contact with each other, and were assured anonymity of their game decisions and questionnaire responses to other participants and the experimenter whom they met in person.

### Trust game

The trust game was played in wave 5 (*N* = 470) between pairs of participants randomly matched from among the 6 to 12 participants who attended the same experimental session. They played the trust game in completely isolated compartment without meeting and interacting in person with other participants. One member of the pair played the role of truster and the other the role of trustee. The truster was provided with JPY 1,000 by the experimenter and decided how much of it to transfer to the trustee, in increments of JPY 100. The transferred money was then tripled and provided to the trustee. The trustee then decided how much of the tripled money to transfer back to the truster. The endowment money of JPY 1,000 was provided only to the truster, and not to the trustee. All participants were told that they would play the game twice, each time with a different partner, and that their role would change. All participants played the truster role in the first game, and the trustee role in the second game. Trustees’ responses in the second game were measured using the strategy method. We used the amounts that the participants transferred as the indicator of their behavioral trust. We used the 470 participants (242 women) who played the trust game in the following analyses.

### General attitudinal trust

Participants responded to a standard trust question, “Do you think most people would try to take advantage of you if they got a chance, or would they not?” in the first and the seventh waves. This question has been routinely used in large-scale surveys such as the General Social Survey and the World Values Survey. The responses were given in a binary form: 0 indicating lack of trust, and 1 indicating trust. We used the mean of the responses in each wave in which participants answered the question in our analysis. In addition to the general attitudinal trust item, we determined the participants’ big five personality traits as defined by the Neo Five-factor Inventory [32] in wave 1 to examine whether personality traits mediate the effects of the rs53576 alleles on behavioral and attitudinal trust.

### Genotyping

Participants’ buccal swabs were collected and preserved in 90% ethanol until DNA extraction. DNA was extracted using the DNeasy Blood & Tissue Kit (QIAGEN, Tokyo, Japan) according to the manufacturer’s protocol. Genotyping of *OXTR* rs53576 was conducted using LAMP Genotyping Series Human *OXTR* (rs53576) (Nippon Gene, Toyama, Japan) by mixing fluorescently labeled LAMP primers and Bst DNA polymerase. The fluorescence level of the reactant was measured by the LAMP-FLP method using a Genie II (Nippon Gene).

## Results

### Genotype distribution

The genotype distribution of the 428 participants was 40.4% AA (*N* = 173), 47.2% AG (N = 202), and 12.4% GG (*N* = 53). This distribution did not significantly differ from the Hardy-Weinberg equilibrium (*χ* ^2^(1) = .558, *p* = .455) and was consistent with those found in previous studies of Asian populations [19,21,30]. Because one of the 428 participants did not participate in the trust game, we used the remaining 427 participants in the following analyses.

### Behavioral trust

**Fig 1:**
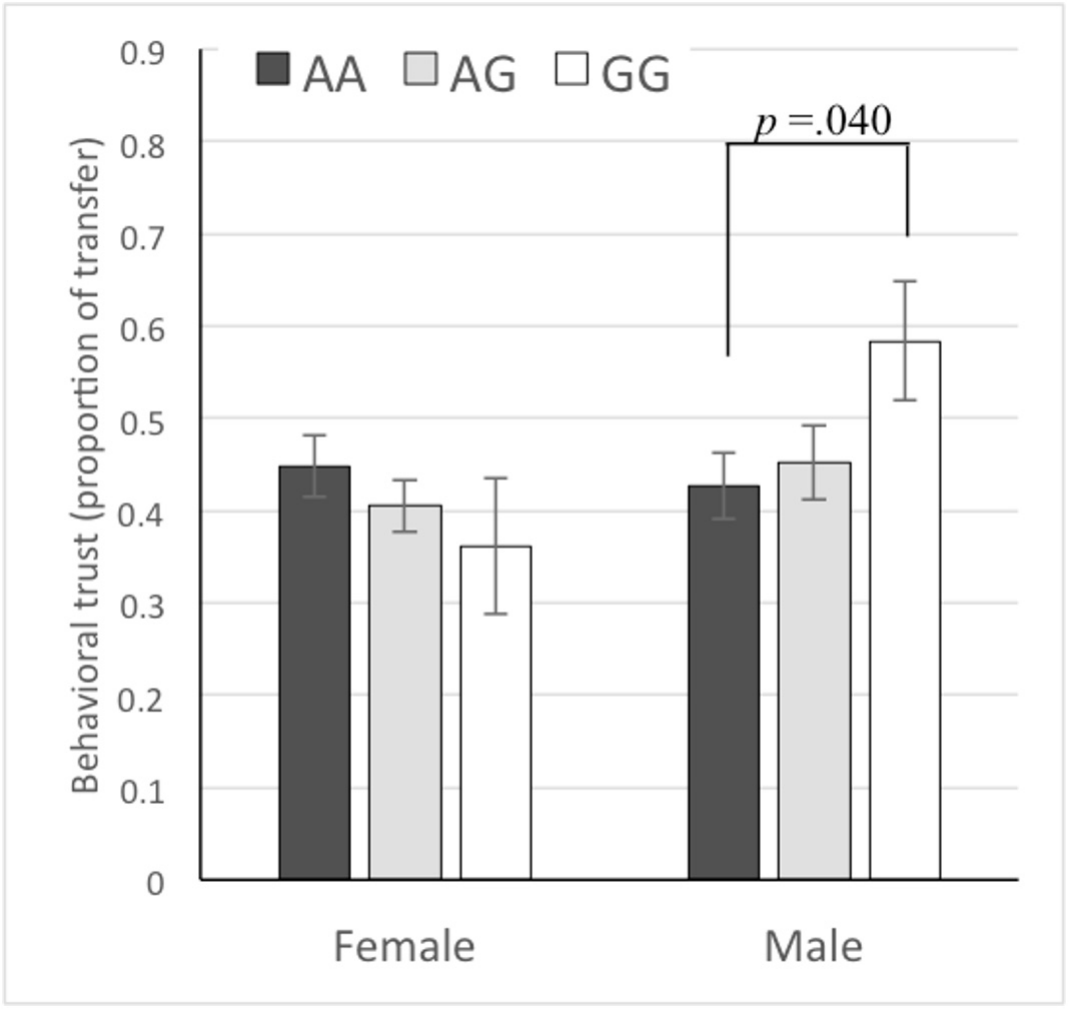
Mean levels of behavioral trust for the three allele types among female and male participants. Error bars show standard error.

The mean proportion of the endowment of JPY 1,000 that the participants transferred as trusters was .434 (SD = .333). Fig 1 shows mean values of the behavioral trust for the three allele types separately for men and women. We conducted a general linear model analysis of behavioral trust (the transfer proportion of the endowment) with two dummy variables for GG (= 1) and AG (= 1; AA defined as the base category), a dummy variable for the participant’s sex (1 = male), and the interaction terms of sex and each of the two allele dummy variables. Because a preliminary analysis revealed a significant effect on behavioral trust of the participants’ age (*r* = .136, *p* = .003), we also added age as a control variable. The effects of age (*F*(1, 421) = 6.76, *p* = .010, *η* ^2^ = .017) and the sex × GG interaction (*F*(1, 421) = 5.12, *p* = .024, *η* ^2^ = .011) were significant. The effects of sex (*F*(1, 421) = 0.11, *p* = .745, *η* ^2^ = .000), GG (*F*(1, 421) = 1.13, *p* = .289, *η* ^2^ = .003), AG (*F*(1, 421) = 0.06, *p* = .813, *η* ^2^ = .000), and the sex × AG interaction (*F*(1, 421) = 0.66, *p* = .416, *η* ^2^ = .002) were not significant. The significant interaction effect suggests that the effect of rs53576 alleles varies between sexes. A separate analysis for male participants showed a significant effect of GG (*F*(1, 207) = 4.26, *p* = .040, *η* ^2^ = .020), whereas the effect of AG was not significant (*F*(1, 207) = 0.13, *p* = .723, *η* ^2^ = .001). Neither GG (*F*(1, 213) = 1.34, *p* = .248, *η* ^2^ = .004) nor AG (*F*(1, 213) = 0.75, *p* = .388, *η* ^2^ = .004) had a significant effect among female participants. No effect was significant in the analysis of the trustee’s behavior, that is, the proportion of the transferred money returned by the trustee. These results indicate a significant difference in behavioral trust between the AA and GG homozygous types among men, whereas the heterozygous AG types did not significantly differ from either of the homozygous types. An additional analysis including the interaction effects of age and the two allele dummy variables indicated that neither of the dummy variables significantly interacted with age.

### General attitudinal trust

**Fig 2:**
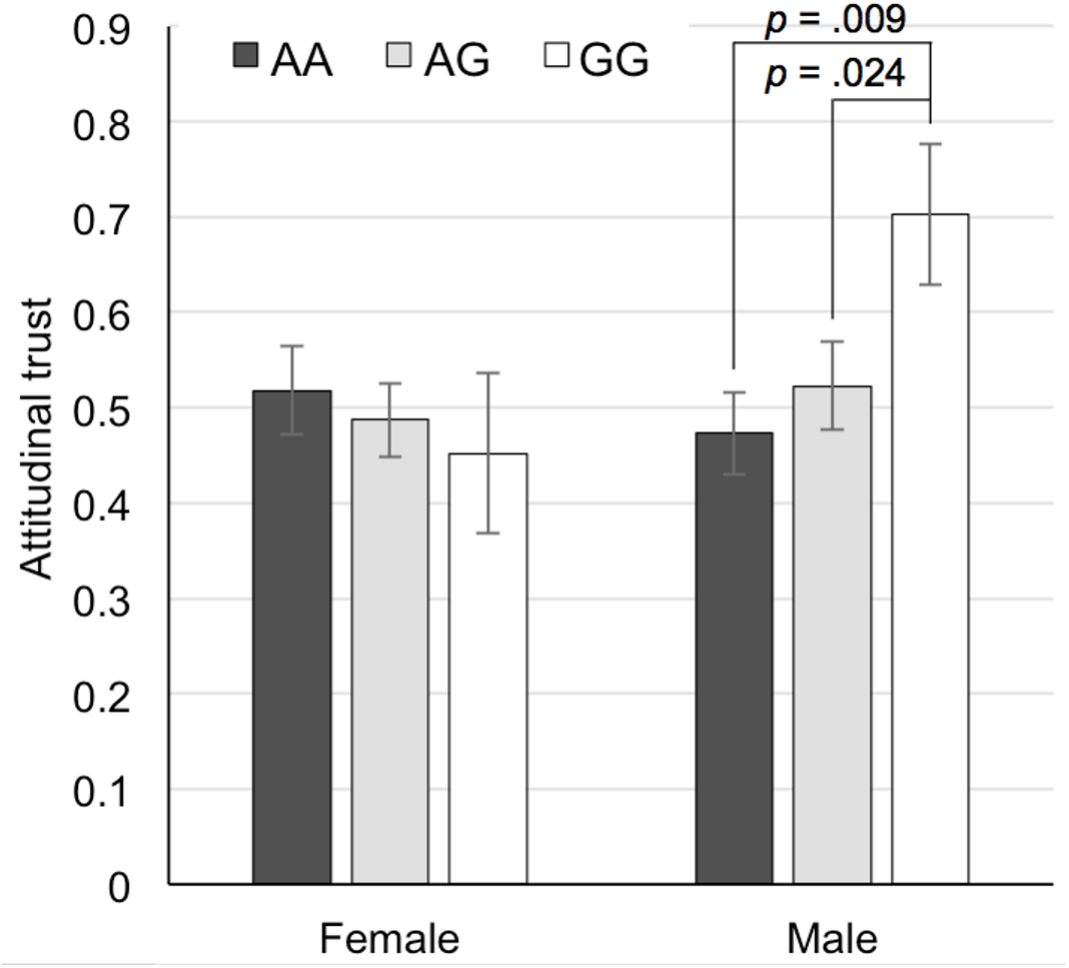
Mean levels of attitudinal trust for the three allele types among female and male participants. Error bars show the standard error.

Our measure of general attitudinal trust positively correlated with behavioral trust observed in the trust game, controlling for age (*r* = .209, *p* < .0001). The correlation was stronger among men (*r* = .287, *p* < .0001) than women (*r* = .121, *p* = .061). Fig 2 displays the differences in the mean levels of general attitudinal trust. The pattern depicted in Fig 2 very much resembles the one shown in Fig 1. As in the analysis of behavioral trust, the effect of age (*F*(1, 421) = 17.84, *p* < .0001, *η* ^2^ = .040), and sex × GG interaction were significant (*F*(1, 421) = 4.91, *p* = .027, *η* ^2^ = .011), and the other effects were not significant. A separate analysis conducted on male participants indicated that the effect of GG (*F*(1, 207) = 6.90, *p* = .009, *η* ^2^ = .031) was significant, and that the effect of AG (*F*(1, 207) = 0.46, *p* = .497, *η* ^2^ = .002) was not. The effect of neither GG (*F*(1, 213) = 0.44, *p* = .506, *η* ^2^ = .002) nor AG (*F*(1, 213) = 0.09, *p* = .766, *η* ^2^ = .000) was significant among women.

In an additional analysis of behavioral trust in which attitudinal trust was controlled, the effect of GG among male participants became non-significant (*F*(1, 206) = 1.92, *p* = .168, *η* ^2^ = .008), whereas the effect of the attitudinal trust was strong (*F*(1, 206) = 16.00, *p* < .0001, *η* ^2^ = .069). A mediation analysis indicates that the effect of the GG allele on behavioral trust was significantly mediated by the attitudinal trust (Sobel’s z = 2.20, *p* = .014, one-tailed). Given that the measurement of attitudinal trust was conducted at least 18 months before and 8 months after the trust game, it is unlikely that the correlation between behavioral and attitudinal trust was generated by the effect of the one-time measurement of behavioral trust in the trust game on the more stable attitudinal trust. The effect of rs53576 genotypes on behavioral trust was likely mediated through effects on the general attitudinal trust.

### Big five personality traits

Table 1 shows the partial correlations of the big five personality traits with behavioral and attitudinal trust, controlling for age. In general, these personality traits were not related to behavioral trust except for a negative correlation among men with conscientiousness. However, some of these traits, particularly agreeableness, extraversion, and neuroticism are related to attitudinal trust for both men and women. When all five personality traits were added as predictors in the regression analysis in which behavioral trust or attitudinal trust was predicted by the two allele dummy variables and age separately for men and women, the effect of GG remained significant on male attitudinal trust (*F*(1, 202) = 5.88, *p* = .016, *η* ^2^ = .022). This result suggests that rs53576 genotypes have a direct bearing on attitudinal trust not mediated by personality traits.

**Table 1:** Partial correlations of the big five personality traits with behavioral and attitudinal trust.

	Behavioral Trust		Attitudinal Trust	
	Female	Male	Female	Male
Agreeableness	.119	.108	.331****	.396****
Extraversion	-.047	.061	.149*	.201**
Neuroticism	-.004	-.005	-.152*	-.221**
Conscientiousness	-.057	-.189**	.095	.015
Openness	.121	.108	.042	.097
GG^1)^	-.054	.130	.001	.159*

^1)^ The standardized regression coefficient for GG in the regression analysis of behavioral and attitudinal trust controlling for the big five personality traits

* *p* < .05

** *p* < .01

**** *p* < .0001

## Discussion

We found that GG men are behaviorally more trusting than the other men, whereas no significant differences were found between the three allele types among women. In fact, GG women tended to be less behaviorally trusting than the other women, although this difference was not statistically significant. This finding replicates the earlier finding of Kruger et al. [8], and provides an explanation for the negative finding by Apicella et al. [25]. Our results suggest that mixing male and female participants obscures the sex-specific trust promoting effect of the GG allele. We also demonstrated that the trust-promoting effect of the GG allele is generalizable to a wider range of age groups, and that the same effect exists in an Asian population in which the distribution of the rs53576 alleles is known to be very different from the Western population [19,21,30].

Another important result is that a similar effect of the GG allele was also observed for general attitudinal trust. Furthermore, the trust-promoting effect of the GG allele among male participants was mediated via enhancement of general attitudinal trust. General attitudinal trust is defined as an expectation of general trustworthiness from other people [33,34], and as such, attitudinal trust *per se* is not a personality trait. However, it is known to be associated with personality traits such as agreeableness and extraversion, in addition to general intelligence [26, 28]. Hiraishi et al. [28] have argued that although general attitudinal trust is not a genetically inherited personality trait in itself, it reacts to other personality traits such as agreeableness and extraversion to adjust for a fitness disadvantage potentially incurred by such personality traits. Our analysis shows that the effect of the GG allele on attitudinal trust is not related to personality traits such as agreeableness or extraversion, and thus does not mediate the GG-attitudinal trust relationship. Although these findings suggest a genuine heritability of general attitudinal trust, more studies are needed to determine the nature of this heritability.

The lack of a relationship between the rs53576 genotype and either behavioral or attitudinal trust among women, which is consistent with the findings of Apicella et al. [25], suggests that additional research is required to provide sufficient explanations. One possible reason for the absence of such a relationship may be ariation in estrogen secretion during the female menstrual cycle. Estrogen is known to modulate oxytocin and its relation to behavior in rats [35] and humans [36]. For example, the higher level of estrogen in pregnancy rats induces a high level of oxytocin emission, and the reduced estrogen level is associated with reduced oxytocin emission [35]. The within-individual variation in the level of oxytocin resulting from the menstrual cycle and pregnancy may thus introduce extra error variance in the between-individual comparison, and may obscure the relationship between the rs53576 genotype and trust

Rilling et al. [37] provide an alternative account of sex differences; they reported sex differences in neural activity following intranasal administration of oxytocin, and further stated that the effect of oxytocin in female participants was not affected by estrogen level. They also reported that administration of oxytocin to men enhanced neural activity of the brain areas rich in oxytocin receptors that play a key role in reward, social bonding, arousal and memory such as the striatum, basal forebrain, insula, amygdala and hippocampus. However, they found that administration of oxytocin to women either decreased activity or had no effect on those brain regions. They argued that one reason for the sex difference observed in neural activity following oxytocin administration is a possible non-linear effect of oxytocin level, according to which the neural activity is the highest in the middle range of oxytocin level (dose plus natural base-rate level). Our finding of no or negative effect of GG over AA for both behavioral and attitudinal trust among female participants may be accounted for by the inverted U-shaped effect of oxytocin on trust [37]. Assuming that the oxytocin levels of GG carriers are generally higher than those of AA carriers, and that the oxytocin levels are generally higher in women than in men, the oxytocin level is expected to be lowest in AA men followed by GG men, and highest in GG women followed by AA women. This results in an inverted U-shaped relationship in which trust is lowest in both extremes of the oxytocin level (AA men and GG women) and the highest in the middle range (GG men and AA women).

Recent studies have also reported sexual differences in the effect of oxytocin administration on the first player’s trusting choice in a sequentially played prisoner’s dilemma game [37], forgiveness of breached trust [38], and moral decision-making [39]. Although the inverted U-shape hypothesis [37] seems to provide a perspective to integrate the findings on sexual differences in *OXTR* genotype studies including the current study and the oxytocin administration studies described above, future investigation of exact mechanisms underlying the relationship among the three factors, i.e., *OXTR* genotype, oxytocin level, and social behavior, is necessary.

Although the cause of the sex difference observed in the relationship between *OXTR* genotype and trust need to be investigated in future studies, the recommendation of Rilling and colleagues [37] against premature generalization of findings obtained in male participants to the female population is worth repeating.

Although we cannot specify the mechanism that connects *OXTR* genotype to male trust, we can speculate on the mediating role of amygdala. The *OXTR* genotype is related to the size of the right amygdala in men but not in women (AA men have a larger right amygdala than GG men) [40], and administration of oxytocin depresses the activity of the right amygdala [41]. Furthermore, patients with amygdala lesions keep trusting even when their trust is breached in a trust game [42]. Given these findings, one possible scenario is that the low level of oxytocin and smaller amygdala in men with the GG allele makes them insensitive to social stresses associated with the risk of breached trust and thus makes them more willing to trust potential interaction partners. The absence of the relationship between *OXTR* genotype and amygdala size in women may explain the lack of effect of *OXTR* genotype on trust in women.

In both behavioral and attitudinal trust, either for men or women, the trust level of heterozygous individuals was between that of the two types of homozygous individuals as shown in Figs 1 and 2. Because of the small sample size for a particular homozygous genome (for example, the GG type in Asian populations and the AA type in Western populations), researchers are often forced to combine one homozygous type with the heterozygous type in assessing the effect of *OXTR* genotype on behavior. Our finding showing that both behavioral and attitudinal measures of trust monotonously increased with the number of G alleles supports the use of such a practice. However, researchers should be aware of the potential bias that they may introduce by combining the heterozygous type with one particular homozygous type versus another.

## Supporting Information

S1 Fig Participants’ gender and age. (DOCX)

S2 Fig Participants’ subjective social class. (DOCX)

S3 Fig Participants’ annual income. (DOCX)

S4 Fig Numbers of participants in million yen. (DOCX)

S5 Fig Participants’ OXTR polymorphism of each age and gender. (DOCX)

S1 Table Data used for the analysis reported in the article. (DOCX)
