# Supplementary material for: Polymorphism of the Oxytocin Receptor Gene Modulates Behavioral and Attitudinal Trust among Men but Not Women
Source: PLoS One. 2015 Oct 7;10(10):e0137089. doi: 10.1371/journal.pone.0137089 (PMC4621758; doi:10.1371/journal.pone.0137089)
Supplement: S4 Fig — (DOCX) [file pone.0137089.s004.docx]

**Fig. S4.** Numbers of participants possessing a 4-year college degree
