# Supplementary material for: Polymorphism of the Oxytocin Receptor Gene Modulates Behavioral and Attitudinal Trust among Men but Not Women
Source: PLoS One. 2015 Oct 7;10(10):e0137089. doi: 10.1371/journal.pone.0137089 (PMC4621758; doi:10.1371/journal.pone.0137089)
Supplement: S1 Table — (DOCX) [file pone.0137089.s006.docx]

**Table S1. Data used for the analysis reported in the article**

The data from all 470 participants used in the analysis are provided as a csv file. The variables are included in the following order.

1. ID number

2. Gender (0=female, 1=male)

3. Age, as of December 2012

4.Subjective social class (1=Lower-Lower, 2=Upper-Lower, 3=Lower-Middle, 4=Upper-Middle, 5=Upper)

5. Annual income (1: JPY 0, 2: < 1.5M, 3: < 3M, 4: < 5M, 5: 7M, 6: <10M, 7: 10M+)

6. College Education (0=Less than college graduate, 1=College graduate)

7. Polymorphism of oxytocin receptor (0=AA, 1=AG, 2=GG)

8. The amount of money the participant provided to the opponent in the role of truster

9. The amount of money the participant provided to the opponent in the role of trustee

10. The mean of the responses in first and seventh wave in which participants answered the general attitudinal trust question

11. Subscale Neuroticism of the Neo Five-factor Inventory

12. Subscale Extraversion of the Neo Five-factor Inventory

13. Subscale Openness of the Neo Five-factor Inventory

14. Subscale Agreeableness of the Neo Five-factor Inventory

15. Subscale Conscientiousness of the Neo Five-factor Inventory

| 10052 | 0 | 57 | 1 | 2 | 0 | 1 | 300 | 0.4 | 1 | 3.17 | 3.58 | 3.50 | 3.67 | 3.50 |
| --- | --- | --- | --- | --- | --- | --- | --- | --- | --- | --- | --- | --- | --- | --- |
| 10053 | 1 | 35 | 2 | 4 | 1 | . | 500 | 0.5 | 0 | 3.17 | 2.17 | 2.58 | 3.08 | 2.67 |
| 10056 | 0 | 46 | 4 | 3 | 1 | 1 | 600 | 0.48 | 0.5 | 2.00 | 3.92 | 2.83 | 3.83 | 3.75 |
| 10057 | 1 | 30 | 1 | 3 | 0 | 1 | 0 | 0.02 | 0 | 4.83 | 1.83 | 3.42 | 2.58 | 2.58 |
| 10059 | 0 | 31 | 3 | 2 | 1 | 1 | 200 | 0 | 0.5 | 3.42 | 3.17 | 3.17 | 3.50 | 2.58 |
| 10068 | 0 | 35 | 1 | 2 | 0 | 1 | 400 | 0.14 | 0 | 4.00 | 2.58 | 3.25 | 2.92 | 2.08 |
| 10071 | 1 | 40 | 3 | 3 | 0 | 2 | 500 | 0.5 | 1 | 3.67 | 3.42 | 3.92 | 3.25 | 3.42 |
| 10072 | 0 | 36 | 3 | 3 | 1 | 1 | 300 | 0.22 | 1 | 3.75 | 2.50 | 3.58 | 3.42 | 3.33 |
| 10075 | 1 | 56 | 4 | 3 | 1 | 0 | 500 | 0 | 1 | 2.83 | 1.92 | 3.75 | 3.08 | 3.58 |
| 10076 | 0 | 22 | 2 | 2 | 0 | 1 | 500 | 0.48 | 0.5 | 4.58 | 3.25 | 3.75 | 3.33 | 3.42 |
| 10077 | 1 | 59 | 3 | 3 | 1 | 1 | 1000 | 0.56 | 1 | 2.75 | 3.33 | 3.50 | 3.75 | 3.58 |
| 10082 | 1 | 28 | 2 | 3 | 1 | 2 | 1000 | 0.44 | 0 | 4.42 | 2.42 | 3.67 | 3.42 | 2.17 |
| 10083 | 1 | 54 | 4 | 6 | 0 | 1 | 500 | 0.4 | 0.5 | 3.33 | 3.83 | 3.25 | 3.67 | 3.58 |
| 10087 | 0 | 50 | 1 | 2 | 0 | 0 | 500 | 0.5 | 1 | 3.33 | 3.17 | 3.33 | 4.33 | 3.17 |
| 10088 | 1 | 30 | 3 | 4 | 0 | 2 | 1000 | 0.5 | 1 | 3.17 | 3.58 | 3.42 | 3.83 | 3.08 |
| 10091 | 1 | 20 | 4 | 2 | 1 | 0 | 0 | 0 | 0.5 | 4.25 | 1.83 | 4.08 | 2.67 | 3.25 |
| 10094 | 0 | 24 | 3 | 3 | 1 | 1 | 500 | 0.5 | 0 | 4.42 | 2.33 | 3.75 | 3.83 | 2.83 |
| 10095 | 1 | 29 | 2 | 4 | 1 | 1 | 1000 | 0.3 | 1 | 2.42 | 3.08 | 3.67 | 3.08 | 4.08 |
| 10109 | 0 | 36 | 4 | 4 | 0 | . | 500 | 0.46 | 1 | 3.92 | 3.50 | 3.33 | 3.25 | 3.08 |
| 10111 | 1 | 54 | 3 | 6 | 1 | 1 | 1000 | 0.4 | 1 | 3.75 | 2.42 | 4.42 | 3.83 | 3.50 |
| 10112 | 1 | 53 | 3 | 6 | 1 | 2 | 1000 | 0.5 | 1 | 3.42 | 3.42 | 3.92 | 3.58 | 3.58 |
| 10113 | 0 | 56 | 3 | 2 | 0 | 0 | 500 | 0.42 | 1 | 2.58 | 3.75 | 4.17 | 4.08 | 3.67 |
| 10117 | 0 | 37 | 4 | 2 | 1 | 1 | 200 | 0.4 | 1 | 3.42 | 2.33 | 3.33 | 3.42 | 3.08 |
| 10118 | 0 | 54 | 2 | 2 | 0 | 0 | 600 | 0.46 | 1 | 3.17 | 3.33 | 3.33 | 3.75 | 3.67 |
| 10119 | 1 | 27 | 4 | 4 | 1 | . | 0 | 0 | 0 | 3.17 | 3.25 | 3.83 | 3.33 | 3.00 |
| 10123 | 1 | 58 | 3 | 5 | 1 | 1 | 500 | 0.32 | 0 | 3.75 | 2.50 | 3.42 | 3.50 | 3.17 |
| 10125 | 0 | 24 | 4 | 4 | 1 | 2 | 0 | 0.2 | 0 | 4.67 | 2.00 | 4.00 | 2.67 | 2.42 |
| 10126 | 0 | 51 | 4 | 2 | 1 | 1 | 400 | 0.32 | 1 | 4.08 | 3.67 | 3.50 | 3.92 | 3.75 |
| 10127 | 1 | 33 | 4 | 6 | 1 | 1 | 0 | 0.3 | 1 | 3.33 | 3.42 | 3.83 | 4.08 | 3.08 |
| 10129 | 0 | 55 | 2 | 3 | 1 | 1 | 200 | 0 | 0.5 | 4.67 | 1.25 | 4.00 | 3.83 | 3.33 |
| 10130 | 0 | 37 | 3 | 2 | 1 | 0 | 500 | 0.4 | 0 | 3.58 | 3.42 | 3.83 | 3.58 | 3.42 |
| 10131 | 0 | 27 | 2 | 2 | 0 | 0 | 600 | 0.5 | 1 | 4.17 | 3.00 | 4.42 | 4.08 | 3.92 |
| 10132 | 0 | 38 | 3 | 3 | 1 | 1 | 300 | 0.3 | 0 | 2.92 | 3.50 | 4.00 | 3.67 | 3.58 |
| 10133 | 0 | 57 | 3 | 3 | 1 | . | 1000 | 0.44 | 0 | 3.00 | 2.50 | 3.83 | 4.33 | 3.33 |
| 10136 | 0 | 45 | 4 | 1 | 0 | 0 | 1000 | 0.5 | 1 | 3.25 | 1.92 | 3.50 | 4.17 | 3.42 |
| 10138 | 1 | 29 | 3 | 4 | 1 | 0 | 200 | 0.3 | 0.5 | 2.58 | 3.33 | 4.42 | 3.25 | 3.42 |
| 10140 | 0 | 45 | 2 | 2 | 0 | 1 | 0 | 0 | 0.5 | 3.83 | 2.75 | 3.42 | 3.00 | 2.83 |
| 10143 | 1 | 32 | 2 | 4 | 0 | . | 1000 | 0.4 | 1 | 4.08 | 3.25 | 4.00 | 2.67 | 2.25 |
| 10144 | 1 | 36 | 4 | 4 | 0 | 1 | 1000 | 1 | 1 | 3.00 | 2.67 | 3.58 | 3.75 | 2.67 |
| 10147 | 0 | 24 | 3 | 2 | 1 | 2 | 200 | 0.26 | 0.5 | 3.50 | 2.67 | 4.17 | 3.00 | 3.33 |
| 10152 | 0 | 31 | 3 | 3 | 1 | 0 | 300 | 0.4 | 0 | 3.08 | 3.75 | 3.58 | 3.92 | 3.42 |
| 10155 | 0 | 38 | 4 | 3 | 1 | 0 | 500 | 0.5 | 0 | 3.42 | 2.92 | 3.42 | 3.83 | 3.33 |
| 10159 | 1 | 59 | 4 | 4 | 0 | 0 | 200 | 0.44 | 0.5 | 2.92 | 3.25 | 3.25 | 3.17 | 3.50 |
| 10161 | 1 | 55 | 3 | 7 | 1 | 1 | 500 | 0.56 | 0.5 | 4.08 | 3.33 | 4.42 | 3.58 | 3.42 |
| 10163 | 0 | 36 | 3 | 3 | 0 | . | 0 | 0.5 | 0 | 3.75 | 2.25 | 3.50 | 3.75 | 2.67 |
| 10165 | 0 | 30 | 3 | 2 | 0 | 0 | 400 | 0.4 | 0 | 2.75 | 3.25 | 3.17 | 3.58 | 3.33 |
| 10168 | 0 | 49 | 4 | 5 | 1 | 1 | 800 | 0.5 | 0 | 4.33 | 1.67 | 4.42 | 3.58 | 2.92 |
| 10170 | 0 | 53 | 4 | 2 | 0 | 2 | 0 | 0 | 1 | 3.42 | 2.92 | 3.75 | 3.92 | 3.33 |
| 10174 | 0 | 42 | 3 | 4 | 1 | 0 | 200 | 0.46 | 1 | 4.33 | 2.08 | 4.50 | 3.75 | 4.08 |
| 10183 | 0 | 31 | 2 | 3 | 1 | 0 | 800 | 0.46 | 0 | 4.00 | 2.75 | 3.67 | 3.17 | 3.17 |
| 10189 | 1 | 40 | 2 | 3 | 1 | 1 | 500 | 0.56 | 0.5 | 3.08 | 3.08 | 4.00 | 3.58 | 3.33 |
| 10194 | 0 | 35 | 3 | 3 | 0 | 0 | 300 | 0.36 | 0.5 | 4.08 | 2.25 | 4.17 | 3.25 | 3.08 |
| 10199 | 0 | 39 | 3 | 2 | 0 | 0 | 1000 | 0.44 | 0 | 4.33 | 3.25 | 3.50 | 3.00 | 2.58 |
| 10213 | 0 | 24 | 2 | 2 | 1 | . | 1000 | 0.46 | 1 | 2.50 | 3.92 | 3.33 | 3.92 | 3.42 |
| 10217 | 1 | 33 | 2 | 3 | 0 | 2 | 600 | 0 | 0 | 2.75 | 3.50 | 2.83 | 3.50 | 3.17 |
| 10221 | 0 | 37 | 2 | 3 | 1 | 0 | 500 | 0.5 | 0 | 4.42 | 2.92 | 3.67 | 2.83 | 2.58 |
| 10222 | 1 | 56 | 2 | 5 | 1 | . | 400 | 0.5 | 0 | 3.58 | 3.17 | 3.92 | 3.50 | 4.17 |
| 10223 | 1 | 28 | 2 | 4 | 1 | 1 | 0 | 0 | 0 | 4.25 | 3.75 | 3.17 | 3.25 | 2.33 |
| 10225 | 0 | 39 | 3 | 2 | 0 | 0 | 100 | 0.1 | 0.5 | 3.33 | 2.50 | 3.08 | 3.83 | 3.00 |
| 10227 | 0 | 35 | 3 | 2 | 0 | 1 | 0 | 0 | 0.5 | 3.42 | 2.75 | 3.75 | 3.33 | 3.00 |
| 10228 | 0 | 37 | 4 | 3 | 1 | 0 | 300 | 0.4 | 1 | 2.42 | 3.75 | 3.58 | 3.75 | 3.33 |
| 10231 | 1 | 56 | 5 | 6 | 1 | 2 | 500 | 0.5 | 0.5 | 3.75 | 3.42 | 3.67 | 3.58 | 3.50 |
| 10232 | 0 | 44 | 4 | 2 | 1 | 0 | 500 | 1 | 1 | 2.83 | 2.67 | 3.75 | 3.92 | 3.25 |
| 10235 | 0 | 44 | 3 | 4 | 1 | 1 | 400 | 0.46 | 1 | 3.42 | 3.17 | 4.00 | 3.75 | 4.00 |
| 10237 | 0 | 52 | 4 | 2 | 1 | 1 | 500 | 0.46 | 1 | 2.50 | 3.83 | 3.42 | 3.67 | 3.50 |
| 10238 | 1 | 31 | 2 | 3 | 0 | 0 | 1000 | 0.5 | 1 | 3.50 | 2.67 | 4.42 | 3.58 | 3.08 |
| 10241 | 0 | 44 | 2 | 3 | 1 | 1 | 900 | 0.42 | 1 | 2.75 | 3.75 | 3.00 | 3.92 | 3.75 |
| 10244 | 0 | 33 | 3 | 2 | 0 | 0 | 500 | 0.48 | 1 | 3.92 | 2.42 | 3.92 | 3.75 | 2.58 |
| 10247 | 0 | 42 | 4 | 3 | 1 | 1 | 300 | 0.48 | 0 | 3.08 | 2.50 | 3.08 | 3.75 | 3.42 |
| 10249 | 0 | 37 | 3 | 2 | 1 | 1 | 800 | 0.36 | 1 | 3.92 | 3.00 | 3.83 | 3.58 | 3.08 |
| 10252 | 1 | 28 | 4 | 5 | 1 | 0 | 0 | 0 | 1 | 3.08 | 3.67 | 4.50 | 3.00 | 3.83 |
| 10254 | 0 | 50 | 4 | 2 | 1 | 1 | 0 | 0 | 0 | 3.33 | 2.25 | 3.75 | 3.42 | 3.67 |
| 10260 | 0 | 40 | 3 | 2 | 1 | 0 | 200 | 0.06 | 1 | 2.92 | 2.58 | 3.33 | 3.42 | 2.73 |
| 10264 | 0 | 36 | 3 | 2 | 0 | 1 | 500 | 0.5 | 0.5 | 3.33 | 3.08 | 3.33 | 3.83 | 2.50 |
| 10267 | 1 | 47 | 3 | 5 | 1 | 1 | 1000 | 0.46 | 1 | 3.00 | 2.58 | 3.50 | 3.50 | 3.33 |
| 10271 | 1 | 51 | 3 | 6 | 1 | 2 | 200 | 0.4 | 1 | 3.08 | 2.83 | 3.36 | 3.67 | 3.75 |
| 10275 | 0 | 37 | 3 | 2 | 0 | 1 | 200 | 0.42 | 0.5 | 4.08 | 3.33 | 3.50 | 3.75 | 3.50 |
| 10277 | 1 | 23 | 3 | 1 | 0 | 0 | 0 | 0 | 0 | 3.67 | 2.33 | 4.08 | 2.75 | 3.00 |
| 10278 | 0 | 47 | 3 | 3 | 0 | 0 | 500 | 0.36 | 0.5 | 2.92 | 4.00 | 3.58 | 4.00 | 3.58 |
| 10283 | 1 | 50 | 4 | 6 | 1 | 2 | 300 | 0.18 | 1 | 3.33 | 3.17 | 4.33 | 4.17 | 3.33 |
| 10284 | 0 | 46 | 3 | 2 | 1 | 0 | 300 | 0.5 | 0 | 3.92 | 3.25 | 3.33 | 3.75 | 3.67 |
| 10285 | 1 | 40 | 3 | 5 | 1 | 1 | 0 | 0 | 0 | 3.17 | 2.92 | 3.92 | 3.08 | 3.08 |
| 10287 | 1 | 48 | 4 | 6 | 1 | 2 | 1000 | 0.42 | 1 | 2.58 | 3.83 | 3.58 | 3.67 | 3.25 |
| 10290 | 1 | 52 | 4 | 7 | 1 | 0 | 800 | 0.3 | 0.5 | 2.92 | 3.42 | 4.00 | 3.25 | 2.75 |
| 10293 | 0 | 58 | 4 | 2 | 1 | 2 | 1000 | 0.4 | 0 | 2.17 | 3.42 | 3.25 | 3.42 | 2.50 |
| 10295 | 1 | 31 | 4 | 6 | 1 | 1 | 500 | 0.16 | 0.5 | 2.50 | 4.08 | 3.83 | 3.92 | 4.33 |
| 10299 | 0 | 27 | 3 | 2 | 1 | 2 | 0 | 0.18 | 0 | 3.92 | 3.50 | 4.00 | 3.33 | 2.83 |
| 10300 | 0 | 48 | 3 | 2 | 1 | 0 | 1000 | 0.38 | 0.5 | 3.42 | 2.83 | 3.83 | 3.92 | 3.58 |
| 10303 | 1 | 59 | 4 | 6 | 1 | 2 | 1000 | 0.5 | 1 | 2.50 | 3.83 | 3.92 | 4.00 | 3.92 |
| 10305 | 0 | 57 | 3 | 2 | 1 | 0 | 500 | 0.38 | 1 | 2.33 | 3.00 | 3.75 | 4.00 | 3.50 |
| 10308 | 0 | 54 | 4 | 2 | 0 | 0 | 400 | 0.42 | 0 | 3.58 | 3.50 | 3.83 | 4.00 | 3.25 |
| 10309 | 0 | 28 | 2 | 2 | 1 | . | 100 | 0.1 | 1 | 4.42 | 4.00 | 3.92 | 3.83 | 3.25 |
| 10312 | 0 | 43 | 2 | 2 | 0 | 0 | 0 | 0 | 0 | 3.83 | 2.75 | 4.42 | 3.50 | 3.08 |
| 10318 | 1 | 47 | 2 | 5 | 0 | 1 | 1000 | 0.5 | 1 | 2.50 | 3.50 | 3.33 | 3.17 | 3.25 |
| 10322 | 0 | 30 | 3 | 2 | 0 | 1 | 0 | 0 | 0 | 4.25 | 1.83 | 4.25 | 3.42 | 2.92 |
| 10326 | 0 | 33 | 4 | 2 | 1 | 1 | 1000 | 0.48 | 1 | 2.83 | 3.33 | 4.08 | 4.00 | 3.42 |
| 10328 | 0 | 42 | 3 | 2 | 1 | . | 300 | 0.36 | 0 | 3.50 | 3.08 | 3.67 | 3.75 | 3.17 |
| 10329 | 0 | 50 | 4 | 2 | 0 | 0 | 200 | 0.44 | 0.5 | 2.25 | 4.08 | 3.17 | 3.92 | 3.42 |
| 10331 | 0 | 42 | 3 | 2 | 0 | 1 | 500 | 0.5 | 1 | 2.83 | 2.17 | 2.75 | 3.33 | 3.08 |
| 10337 | 1 | 50 | 2 | 3 | 1 | 0 | 500 | 0.5 | 1 | 2.25 | 4.08 | 3.75 | 3.75 | 3.67 |
| 10342 | 0 | 35 | 2 | 3 | 1 | 0 | 200 | 0.1 | 0 | 3.67 | 3.00 | 4.25 | 3.33 | 3.00 |
| 10343 | 1 | 25 | 3 | 3 | 0 | 0 | 300 | 0.34 | 0 | 3.92 | 2.17 | 2.75 | 3.50 | 2.33 |
| 10344 | 1 | 51 | 1 | 3 | 1 | 1 | 300 | 0 | 1 | 3.92 | 2.00 | 3.50 | 4.17 | 2.25 |
| 10346 | 1 | 29 | 3 | 4 | 1 | 2 | 200 | 0.32 | 0 | 3.50 | 3.17 | 3.17 | 4.17 | 2.92 |
| 10350 | 0 | 48 | 4 | 4 | 0 | 0 | 400 | 0.38 | 0.5 | 3.17 | 3.25 | 3.75 | 3.67 | 4.08 |
| 10356 | 0 | 50 | 3 | 2 | 1 | 1 | 200 | 0.32 | 0 | 2.42 | 3.33 | 3.42 | 3.75 | 4.00 |
| 10361 | 0 | 51 | 3 | 3 | 0 | 1 | 700 | 0.6 | 1 | 2.75 | 3.33 | 3.67 | 3.58 | 3.33 |
| 10375 | 0 | 39 | 2 | 2 | 0 | 1 | 1000 | 0.5 | 0 | 4.25 | 2.33 | 4.00 | 3.25 | 3.25 |
| 10377 | 1 | 24 | 4 | 4 | 0 | 0 | 700 | 0.4 | 0 | 3.83 | 3.42 | 4.08 | 3.00 | 3.08 |
| 10378 | 0 | 37 | 4 | 1 | 1 | 1 | 600 | 0.24 | 0.5 | 2.42 | 2.83 | 3.75 | 4.00 | 2.83 |
| 10381 | 0 | 36 | 3 | 3 | 0 | 1 | 200 | 0 | 0 | 2.75 | 3.33 | 3.33 | 3.08 | 3.42 |
| 10382 | 1 | 33 | 2 | 4 | 0 | 0 | 200 | 0.38 | 0 | 3.00 | 3.25 | 4.58 | 2.33 | 3.25 |
| 10386 | 0 | 29 | 1 | 3 | 1 | 2 | 200 | 0 | 0 | 4.33 | 3.08 | 4.00 | 3.00 | 2.58 |
| 10388 | 0 | 24 | 3 | 3 | 1 | 1 | 600 | 0.44 | 0 | 4.42 | 2.42 | 4.58 | 2.92 | 2.58 |
| 10391 | 0 | 30 | 3 | 3 | 1 | 2 | 1000 | 0.4 | 0.5 | 4.00 | 3.42 | 4.00 | 3.92 | 2.92 |
| 10393 | 1 | 50 | 3 | 6 | 1 | 0 | 600 | 0.3 | 1 | 2.33 | 2.92 | 3.33 | 3.25 | 3.33 |
| 10394 | 1 | 48 | 4 | 5 | 0 | 1 | 1000 | 0.9 | 0.5 | 2.67 | 3.67 | 4.17 | 3.92 | 4.00 |
| 10395 | 0 | 38 | 4 | 2 | 1 | 0 | 500 | 0.5 | 1 | 3.92 | 3.42 | 3.92 | 3.83 | 3.00 |
| 10398 | 0 | 46 | 4 | 1 | 1 | . | 200 | 0.4 | 1 | 4.17 | 3.42 | 4.17 | 3.42 | 3.75 |
| 10399 | 0 | 50 | 3 | 2 | 1 | . | 300 | 0.44 | 0 | 3.50 | 2.75 | 3.17 | 4.00 | 3.42 |
| 10404 | 1 | 29 | 2 | 4 | 1 | 1 | 500 | 0.5 | 0.5 | 3.67 | 2.75 | 3.25 | 3.25 | 2.83 |
| 10409 | 1 | 52 | 3 | 5 | 0 | 0 | 100 | 0.32 | 1 | 3.00 | 3.67 | 4.50 | 3.25 | 2.92 |
| 10410 | 0 | 35 | 1 | 3 | 0 | 1 | 0 | 0 | 0.5 | 4.92 | 2.08 | 3.92 | 2.50 | 3.67 |
| 10412 | 1 | 52 | 3 | 5 | 1 | 1 | 1000 | 0.46 | 1 | 3.33 | 3.08 | 3.92 | 3.67 | 2.83 |
| 10413 | 0 | 28 | 3 | 4 | 1 | . | 300 | 0.4 | 0 | 3.17 | 3.08 | 4.58 | 4.33 | 4.00 |
| 10418 | 0 | 47 | 3 | 2 | 0 | 1 | 500 | 0.5 | 1 | 4.25 | 3.08 | 4.00 | 3.58 | 3.83 |
| 10422 | 0 | 28 | 3 | 4 | 1 | 1 | 1000 | 1 | 0 | 2.33 | 3.42 | 4.33 | 3.25 | 3.83 |
| 10423 | 1 | 51 | 4 | 4 | 1 | 0 | 300 | 0.4 | 1 | 3.75 | 2.33 | 4.08 | 3.67 | 3.25 |
| 10424 | 1 | 53 | 4 | 7 | 1 | 1 | 500 | 0.5 | 1 | 3.17 | 2.17 | 3.25 | 3.92 | 3.83 |
| 10432 | 0 | 43 | 3 | 2 | 0 | 1 | 500 | 0.5 | 1 | 2.75 | 4.00 | 3.75 | 3.75 | 4.00 |
| 10433 | 0 | 51 | 3 | 2 | 0 | 0 | 300 | 0.42 | 0 | 2.58 | 2.33 | 3.75 | 3.83 | 2.67 |
| 10434 | 0 | 48 | 4 | 5 | 1 | 1 | 400 | 0.44 | 0 | 2.17 | 3.67 | 3.67 | 4.25 | 3.83 |
| 10436 | 0 | 56 | 4 | 2 | 0 | 2 | 500 | 0.5 | 0.5 | 3.17 | 2.75 | 3.83 | 3.58 | 3.08 |
| 10437 | 0 | 46 | 3 | 2 | 1 | 0 | 0 | 0.5 | 0 | 2.58 | 2.75 | 4.08 | 3.42 | 4.08 |
| 10440 | 0 | 58 | 3 | 3 | 0 | 0 | 1000 | 0.4 | 0 | 3.67 | 2.67 | 3.50 | 3.50 | 3.42 |
| 10445 | 0 | 34 | 3 | 4 | 1 | 0 | 0 | 0 | 0.5 | 4.25 | 3.00 | 2.92 | 3.17 | 3.33 |
| 10446 | 0 | 52 | 4 | 6 | 0 | 0 | 600 | 0.36 | 0.5 | 3.08 | 3.58 | 3.83 | 3.75 | 3.25 |
| 10447 | 1 | 39 | 3 | 4 | 1 | 2 | 600 | 0.7 | 1 | 2.50 | 3.75 | 4.25 | 4.08 | 2.58 |
| 10449 | 1 | 49 | 3 | 3 | 1 | 1 | 100 | 0.4 | 1 | 3.67 | 3.25 | 3.25 | 4.00 | 3.17 |
| 10451 | 1 | 54 | 4 | 7 | 1 | 2 | 500 | 0.4 | 1 | 2.67 | 2.25 | 3.58 | 4.08 | 3.83 |
| 10456 | 1 | 45 | 3 | 4 | 1 | 2 | 200 | 0.4 | 0.5 | 3.67 | 2.83 | 4.33 | 3.50 | 3.58 |
| 10458 | 1 | 42 | 2 | 4 | 1 | 1 | 500 | 0.1 | 0.5 | 3.75 | 3.17 | 3.25 | 3.67 | 2.75 |
| 10460 | 1 | 45 | 3 | 4 | 0 | 1 | 300 | 0.42 | 0.5 | 2.58 | 3.00 | 3.00 | 3.67 | 3.42 |
| 10464 | 1 | 55 | 3 | 4 | 1 | 1 | 300 | 0.38 | 0 | 3.42 | 2.75 | 3.67 | 4.00 | 3.42 |
| 10466 | 0 | 41 | 4 | 2 | 1 | 0 | 700 | 0.44 | 1 | 3.67 | 3.08 | 4.42 | 3.75 | 3.00 |
| 10468 | 0 | 57 | 4 |  | 1 | . | 0 | 0 | 1 | 3.00 | 2.75 | 3.17 | 4.08 | 3.58 |
| 10470 | 0 | 34 | 2 | 2 | 1 | 1 | 600 | 0.42 | 1 | 3.42 | 2.50 | 3.17 | 3.58 | 2.58 |
| 10472 | 0 | 34 | 3 | 4 | 1 | . | 400 | 0.5 | 1 | 3.92 | 2.83 | 4.08 | 3.42 | 3.17 |
| 10473 | 0 | 50 | 2 | 3 | 0 | 0 | 400 | 0.4 | 0 | 3.58 | 2.50 | 3.33 | 3.92 | 2.67 |
| 10475 | 1 | 19 | 1 | 3 | 0 | . | 0 | 0 | 1 | 2.75 | 4.08 | 4.50 | 4.00 | 3.83 |
| 10476 | 0 | 29 | 1 | 3 | 0 | 0 | 0 | 0.5 | 0.5 | 3.83 | 3.92 | 4.17 | 3.42 | 3.25 |
| 10477 | 0 | 56 | 3 | 4 | 0 | 0 | 0 | 0 | 1 | 2.92 | 3.08 | 3.42 | 3.67 | 3.33 |
| 10478 | 1 | 42 | 2 | 3 | 1 | 2 | 200 | 0.5 | 0.5 | 3.50 | 2.92 | 3.75 | 3.17 | 3.33 |
| 10480 | 0 | 52 | 4 | 2 | 1 | 0 | 100 | 0.34 | 0 | 4.08 | 3.75 | 4.33 | 3.50 | 2.33 |
| 10481 | 0 | 35 | 3 | 3 | 0 | 0 | 500 | 0.5 | 1 | 3.00 | 3.17 | 3.75 | 3.83 | 3.25 |
| 10483 | 0 | 47 | 3 | 5 | 1 | 1 | 500 | 0.48 | 0.5 | 3.25 | 3.17 | 3.67 | 3.17 | 3.17 |
| 10486 | 0 | 53 | 3 | 2 | 0 | 2 | 100 | 0 | 1 | 3.50 | 2.75 | 4.17 | 3.83 | 4.08 |
| 10488 | 0 | 23 | 1 | 2 | 1 | 0 | 1000 | 0.1 | 0.5 | 3.17 | 4.08 | 4.58 | 3.75 | 3.08 |
| 10491 | 1 | 35 | 3 | 4 | 0 | 2 | 1000 | 0.5 | 1 | 3.00 | 3.25 | 3.83 | 3.58 | 3.83 |
| 10492 | 0 | 43 | 3 | 4 | 0 | 1 | 0 | 0 | 0 | 3.75 | 2.83 | 3.42 | 3.25 | 3.83 |
| 10494 | 0 | 55 | 3 | 4 | 0 | 1 | 600 | 0.48 | 1 | 2.33 | 3.92 | 4.25 | 4.83 | 4.50 |
| 10496 | 0 | 47 | 3 | 2 | 1 | 0 | 200 | 0.22 | 0.5 | 3.33 | 2.92 | 3.50 | 3.00 | 3.50 |
| 10497 | 1 | 34 | 3 | 4 | 1 | 0 | 500 | 0.44 | 0 | 3.92 | 2.08 | 3.25 | 3.33 | 3.42 |
| 10498 | 0 | 24 | 3 | 4 | 1 | 1 | 0 | 0 | 1 | 3.67 | 3.17 | 4.08 | 3.50 | 3.17 |
| 10499 | 1 | 59 | 3 | 6 | 0 | 0 | 300 | 0.1 | 0.5 | 3.17 | 3.08 | 3.42 | 4.00 | 3.58 |
| 10501 | 1 | 51 | 3 | 3 | 1 | 0 | 100 | 0.3 | 0.5 | 3.58 | 3.58 | 3.50 | 3.58 | 3.08 |
| 10503 | 0 | 58 | 4 | 2 | 0 | 0 | 400 | 0.5 | 0.5 | 3.25 | 3.42 | 3.33 | 3.67 | 3.00 |
| 10504 | 1 | 36 | 2 | 4 | 1 | 1 | 1000 | 0.42 | 1 | 2.67 | 3.50 | 3.08 | 3.42 | 3.25 |
| 10506 | 0 | 50 | 2 | 2 | 0 | 1 | 400 | 0.3 | 0 | 4.00 | 2.92 | 4.17 | 3.17 | 3.42 |
| 10507 | 1 | 23 | 4 |  | 1 | . | 0 | 0 | 0 | 3.17 | 3.00 | 4.08 | 3.92 | 4.00 |
| 10509 | 0 | 50 | 3 | 5 | 0 | . | 500 | 0.46 | 1 | 3.25 | 3.50 | 3.75 | 4.00 | 3.67 |
| 10515 | 0 | 41 | 2 | 3 | 0 | 0 | 1000 | 0.48 | 0.5 | 3.42 | 3.00 | 3.50 | 3.17 | 2.75 |
| 10516 | 1 | 53 | 2 | 5 | 1 | 1 | 600 | 0.36 | 0 | 3.33 | 2.75 | 3.67 | 3.00 | 3.00 |
| 10518 | 0 | 51 | 3 | 2 | 0 | 1 | 1000 | 0.42 | 1 | 3.42 | 3.83 | 4.00 | 3.83 | 3.50 |
| 10521 | 1 | 35 | 3 | 5 | 1 | 1 | 0 | 0.38 | 0 | 3.83 | 3.67 | 3.75 | 3.58 | 3.92 |
| 10525 | 1 | 49 | 3 | 5 | 0 | 0 | 400 | 0.1 | 0 | 2.83 | 3.42 | 3.33 | 3.17 | 3.83 |
| 10529 | 1 | 58 | 3 | 6 | 0 | 1 | 1000 | 0.5 | 1 | 2.92 | 3.00 | 3.17 | 4.08 | 3.75 |
| 10530 | 0 | 40 | 4 | 4 | 1 | . | 0 | 0 | 1 | 3.50 | 4.17 | 2.92 | 4.17 | 3.42 |
| 10533 | 0 | 25 | 1 | 4 | 0 | 1 | 0 | 0.1 | 0.5 | 3.33 | 3.00 | 3.58 | 3.75 | 3.00 |
| 10536 | 1 | 32 | 3 | 4 | 1 | . | 0 | 0 | 0 | 3.33 | 3.00 | 3.33 | 3.42 | 3.00 |
| 10541 | 0 | 32 | 4 | 3 | 0 | . | 500 | 0.4 | 0.5 | 2.58 | 3.17 | 3.58 | 3.92 | 3.92 |
| 10542 | 0 | 23 | 2 | 3 | 1 | 0 | 1000 | 0.48 | 0 | 4.75 | 3.67 | 4.08 | 3.25 | 4.00 |
| 10543 | 0 | 55 | 4 | 2 | 1 | . | 400 | 0.5 | 1 | 3.33 | 3.42 | 3.42 | 3.75 | 3.42 |
| 10544 | 1 | 56 | 4 | 7 | 1 | 0 | 600 | 0.24 | 1 | 2.67 | 3.17 | 4.00 | 4.50 | 4.17 |
| 10545 | 0 | 50 | 3 | 2 | 0 | 0 | 200 | 0.48 | 0.5 | 2.92 | 2.33 | 3.58 | 3.42 | 3.00 |
| 10546 | 0 | 47 | 3 | 6 | 1 | 0 | 1000 | 0.44 | 1 | 2.83 | 2.33 | 3.50 | 3.50 | 3.50 |
| 10547 | 0 | 57 | 4 | 2 | 1 | 2 | 600 | 0.5 | 0.5 | 4.33 | 3.50 | 3.67 | 3.75 | 3.25 |
| 10553 | 1 | 46 | 2 | 4 | 0 | 0 | 500 | 0.44 | 0 | 3.83 | 2.58 | 3.92 | 3.33 | 3.25 |
| 10555 | 1 | 47 | 3 | 5 | 1 | 0 | 200 | 0.28 | 0 | 3.25 | 3.08 | 3.92 | 3.50 | 4.08 |
| 10557 | 1 | 41 | 3 | 4 | 1 | 0 | 500 | 0.5 | 0.5 | 3.17 | 3.00 | 3.75 | 3.67 | 3.08 |
| 10559 | 0 | 36 | 3 | 2 | 1 | 2 | 1000 | 0.3 | 0.5 | 3.42 | 2.75 | 3.25 | 3.83 | 2.92 |
| 10563 | 0 | 46 | 3 | 2 | 0 | 1 | 0 | 0.1 | 1 | 3.25 | 3.67 | 3.75 | 4.33 | 2.75 |
| 10565 | 0 | 44 | 2 | 4 | 0 | . | 200 | 0.5 | 0 | 3.00 | 3.00 | 3.42 | 3.08 | 3.42 |
| 10567 | 0 | 48 | 3 | 5 | 1 | 2 | 100 | 0.22 | 1 | 3.42 | 3.50 | 4.00 | 3.58 | 3.92 |
| 10569 | 0 | 48 | 4 | 1 | 0 | 0 | 500 | 0.5 | 0.5 | 3.33 | 3.58 | 3.67 | 3.75 | 3.42 |
| 10573 | 0 | 39 | 3 | 2 | 1 | 1 | 100 | 0.3 | 1 | 3.33 | 3.42 | 3.58 | 3.50 | 2.75 |
| 10576 | 0 | 21 | 1 | 2 | 1 | . | 600 | 0.5 | 0 | 3.83 | 2.92 | 3.67 | 3.58 | 3.42 |
| 10577 | 0 | 51 | 3 | 4 | 0 | 0 | 600 | 0.42 | 1 | 3.08 | 3.08 | 4.33 | 4.33 | 4.00 |
| 10579 | 1 | 40 | 3 | 4 | 1 | 2 | 0 | 0 | 0 | 2.67 | 2.75 | 4.08 | 2.75 | 3.33 |
| 10582 | 1 | 38 | 2 | 3 | 1 | 0 | 1000 | 0.4 | 1 | 3.75 | 3.00 | 3.00 | 3.75 | 2.67 |
| 10585 | 0 | 51 | 3 | 4 | 1 | 2 | 300 | 0.4 | 1 | 3.00 | 3.50 | 3.33 | 3.42 | 4.08 |
| 10586 | 0 | 37 | 2 | 5 | 0 | 0 | 500 | 0.4 | 0 | 3.08 | 3.17 | 3.50 | 2.75 | 3.42 |
| 10591 | 0 | 50 | 4 | 2 | 1 | 1 | 400 | 0.24 | 0.5 | 3.08 | 3.42 | 2.92 | 3.58 | 3.33 |
| 10593 | 0 | 49 | 4 | 1 | 0 | 1 | 300 | 0.36 | 0.5 | 3.75 | 3.00 | 3.33 | 3.92 | 3.17 |
| 10594 | 0 | 55 | 4 | 5 | 0 | 1 | 500 | 0.5 | 0.5 | 3.83 | 2.50 | 3.58 | 3.92 | 2.83 |
| 10597 | 1 | 37 | 2 | 6 | 1 | 1 | 600 | 0.32 | 1 | 2.58 | 3.58 | 3.75 | 4.33 | 3.17 |
| 10599 | 1 | 35 | 3 | 4 | 0 | 0 | 0 | 0 | 0.5 | 2.42 | 3.42 | 3.17 | 3.92 | 2.00 |
| 10602 | 0 | 45 | 2 | 2 | 0 | 1 | 700 | 0.48 | 1 | 3.92 | 1.92 | 3.83 | 3.17 | 1.83 |
| 10603 | 0 | 39 | 3 | 2 | 0 | 1 | 100 | 0.12 | 0.5 | 2.67 | 2.67 | 2.67 | 3.00 | 2.75 |
| 10605 | 0 | 32 | 4 | 4 | 1 | 0 | 100 | 0.34 | 0.5 | 3.08 | 3.25 | 3.75 | 3.58 | 3.83 |
| 10609 | 0 | 34 | 3 | 2 | 1 | 1 | 0 | 0.14 | 1 | 3.25 | 3.75 | 3.50 | 3.92 | 3.25 |
| 10610 | 1 | 53 | 4 | 6 | 1 | 0 | 500 | 0.28 | 1 | 2.42 | 3.42 | 3.33 | 4.42 | 4.17 |
| 10613 | 0 | 43 | 2 | 4 | 1 | . | 500 | 0.5 | 0 | 3.83 | 2.92 | 3.08 | 4.17 | 3.33 |
| 10617 | 0 | 52 | 3 | 2 | 1 | 1 | 500 | 0.5 | 0.5 | 2.75 | 3.25 | 4.17 | 4.00 | 3.75 |
| 10620 | 1 | 34 | 4 | 5 | 1 | 0 | 800 | 0.62 | 1 | 2.58 | 3.58 | 3.83 | 4.17 | 4.17 |
| 10622 | 0 | 30 | 3 | 3 | 1 | . | 300 | 0 | 1 | 2.50 | 3.25 | 3.67 | 3.92 | 3.50 |
| 10623 | 1 | 46 | 2 | 5 | 1 | 1 | 700 | 0 | 1 | 3.58 | 2.83 | 4.00 | 3.25 | 3.00 |
| 10626 | 0 | 42 | 3 | 2 | 1 | 0 | 300 | 0.42 | 0 | 4.75 | 3.25 | 3.83 | 3.17 | 3.92 |
| 10627 | 1 | 37 | 4 | 6 | 1 | 2 | 1000 | 0.4 | 1 | 3.67 | 2.50 | 4.08 | 3.33 | 2.42 |
| 10629 | 1 | 39 | 2 | 3 | 1 | 0 | 1000 | 0 | 0 | 3.67 | 2.42 | 3.42 | 2.50 | 3.17 |
| 10630 | 0 | 32 | 3 | 2 | 0 | 0 | 300 | 0.3 | 1 | 4.00 | 3.50 | 3.67 | 4.08 | 2.58 |
| 10631 | 0 | 53 | 4 | 2 | 1 | 0 | 500 | 0.5 | 0 | 3.58 | 2.83 | 3.08 | 3.75 | 3.00 |
| 10633 | 0 | 25 | 3 | 3 | 1 | 1 | 500 | 0.5 | 0 | 4.17 | 2.92 | 3.92 | 3.33 | 3.33 |
| 10635 | 1 | 58 | 3 | 4 | 1 | 1 | 1000 | 0.44 | 1 | 2.83 | 4.00 | 3.67 | 3.42 | 3.33 |
| 10641 | 0 | 38 | 2 | 2 | 0 | 1 | 0 | 0 | 0 | 4.17 | 3.83 | 4.25 | 3.50 | 2.75 |
| 10644 | 1 | 35 | 3 | 5 | 1 | 2 | 500 | 0.42 | 1 | 3.00 | 3.42 | 3.17 | 3.92 | 3.67 |
| 10649 | 0 | 47 | 3 | 2 | 0 | 1 | 300 | 0.38 | 0.5 | 3.83 | 2.92 | 3.17 | 4.08 | 3.50 |
| 10651 | 1 | 41 | 3 | 3 | 0 | 2 | 300 | 0.22 | 1 | 3.92 | 3.00 | 3.92 | 3.33 | 3.42 |
| 10654 | 0 | 36 | 2 | 2 | 0 | 1 | 600 | 0.32 | 0.5 | 3.17 | 3.33 | 4.42 | 3.25 | 3.42 |
| 10658 | 0 | 56 | 4 | 4 | 1 | 1 | 500 | 0.76 | 0.5 | 2.67 | 3.42 | 4.00 | 3.92 | 4.33 |
| 10659 | 0 | 57 | 3 | 2 | 0 | 1 | 400 | 0.5 | 0.5 | 3.33 | 2.83 | 3.33 | 3.50 | 3.67 |
| 10660 | 1 | 51 | 3 | 6 | 1 | 0 | 200 | 0.4 | 0.5 | 4.00 | 2.50 | 3.83 | 3.33 | 3.67 |
| 10662 | 0 | 22 | 3 | 2 | 1 | 1 | 0 | 0 | 0 | 3.08 | 3.50 | 3.42 | 2.92 | 4.50 |
| 10663 | 1 | 24 | 2 | 2 | 0 | 0 | 0 | 0 | 0 | 3.67 | 2.58 | 2.33 | 3.67 | 1.67 |
| 10664 | 1 | 51 | 3 | 5 | 1 | 1 | 100 | 0.2 | 0.5 | 2.42 | 2.92 | 3.58 | 3.83 | 3.58 |
| 10667 | 0 | 52 | 2 | 3 | 0 | 1 | 300 | 0.3 | 1 | 3.08 | 3.83 | 4.08 | 3.83 | 3.67 |
| 10668 | 1 | 32 | 3 | 5 | 1 | 1 | 1000 | 0.5 | 1 | 4.50 | 1.67 | 3.58 | 3.92 | 2.08 |
| 10673 | 0 | 33 | 4 | 2 | 1 | 1 | 1000 | 0.5 | 1 | 4.33 | 2.67 | 4.33 | 3.75 | 3.25 |
| 10675 | 0 | 49 | 3 | 3 | 0 | 1 | 300 | 0.5 | 0 | 3.58 | 3.33 | 3.08 | 3.17 | 3.25 |
| 10678 | 0 | 46 | 4 | 5 | 0 | 0 | 300 | 0.3 | 1 | 2.42 | 3.42 | 3.33 | 4.08 | 3.58 |
| 10680 | 0 | 42 | 3 | 2 | 1 | 1 | 500 | 0.4 | 0 | 3.67 | 2.67 | 3.08 | 3.58 | 3.33 |
| 10682 | 0 | 37 | 3 | 3 | 0 | 1 | 1000 | 0.3 | 0 | 3.42 | 3.17 | 3.83 | 3.08 | 3.00 |
| 10687 | 0 | 47 | 4 | 2 | 0 | . | 400 | 0.44 | 0 | 3.25 | 2.92 | 3.42 | 3.33 | 2.17 |
| 10690 | 0 | 47 | 1 | 2 | 0 | 1 | 500 | 0.5 | 0 | 4.00 | 2.50 | 3.08 | 2.50 | 3.00 |
| 10696 | 0 | 44 | 3 | 1 | 0 | 1 | 500 | 0.44 | 1 | 2.25 | 2.58 | 3.00 | 3.92 | 3.25 |
| 10701 | 0 | 54 | 3 | 2 | 0 | 0 | 0 | 0 | 0 | 4.58 | 2.75 | 4.42 | 2.82 | 2.58 |
| 10705 | 1 | 27 | 2 | 4 | 0 | 0 | 500 | 0.06 | 0 | 2.50 | 3.50 | 4.17 | 4.17 | 3.33 |
| 10706 | 0 | 35 | 4 | 4 | 0 | . | 300 | 0.44 | 1 | 4.00 | 2.50 | 2.33 | 2.67 | 3.58 |
| 10708 | 0 | 48 | 3 | 2 | 0 | 2 | 500 | 0.42 | 0.5 | 3.83 | 4.25 | 3.08 | 3.67 | 3.75 |
| 10709 | 0 | 56 | 3 | 2 | 0 | 0 | 500 | 0.5 | 1 | 4.25 | 3.00 | 4.00 | 4.33 | 3.08 |
| 10710 | 0 | 41 | 3 | 2 | 1 | 1 | 1000 | 0.3 | 0 | 3.00 | 3.50 | 3.92 | 4.00 | 3.50 |
| 10711 | 0 | 25 | 3 | 4 | 1 | 1 | 300 | 0.42 | 0.5 | 3.50 | 3.08 | 4.17 | 3.08 | 3.42 |
| 10712 | 1 | 39 | 3 | 4 | 0 | 0 | 0 | 0 | 1 | 3.00 | 2.67 | 3.92 | 4.58 | 2.58 |
| 10715 | 1 | 41 | 3 | 5 | 1 | 1 | 1000 | 0.46 | 0.5 | 3.50 | 2.75 | 3.50 | 3.92 | 3.33 |
| 10718 | 0 | 40 | 4 | 2 | 0 | 1 | 500 | 0.38 | 0.5 | 3.75 | 2.75 | 3.67 | 3.58 | 2.92 |
| 10719 | 0 | 29 | 3 | 1 | 1 | 1 | 0 | 0.2 | 1 | 3.25 | 2.67 | 3.33 | 3.67 | 3.08 |
| 10721 | 0 | 27 | 3 | 2 | 1 | 2 | 700 | 0 | 0.5 | 2.50 | 3.33 | 3.50 | 3.75 | 4.08 |
| 10722 | 1 | 48 | 2 | 5 | 1 | 0 | 200 | 0.1 | 0 | 3.33 | 3.17 | 3.50 | 3.25 | 4.25 |
| 10723 | 0 | 46 | 4 | 2 | 1 | 1 | 100 | 0.42 | 1 | 2.50 | 3.67 | 3.50 | 3.58 | 3.00 |
| 10724 | 0 | 55 | 3 | 2 | 1 | 1 | 500 | 0.5 | 0.5 | 3.50 | 3.25 | 3.75 | 4.00 | 3.92 |
| 10731 | 1 | 45 | 3 | 5 | 0 | 1 | 400 | 0.4 | 0.5 | 2.00 | 3.83 | 2.58 | 4.42 | 2.50 |
| 10733 | 0 | 21 | 4 | 3 | 1 | 1 | 0 | 0 | 0 | 3.17 | 2.50 | 3.42 | 3.17 | 3.67 |
| 10734 | 0 | 42 | 4 | 2 | 1 | . | 500 | 0.5 | 0 | 3.42 | 3.50 | 3.17 | 4.00 | 2.92 |
| 10735 | 1 | 40 | 3 | 6 | 1 | 1 | 400 | 0.54 | 0 | 3.50 | 2.67 | 3.00 | 3.25 | 2.50 |
| 10738 | 0 | 26 | 3 | 2 | 1 | 1 | 500 | 0.16 | 1 | 3.83 | 2.50 | 3.83 | 3.42 | 2.17 |
| 10739 | 1 | 38 | 2 | 4 | 0 | 1 | 300 | 0.4 | 0 | 2.67 | 3.33 | 2.83 | 3.33 | 3.17 |
| 10744 | 1 | 37 | 3 | 2 | 1 | 1 | 100 | 0.4 | 1 | 2.92 | 3.33 | 2.83 | 3.67 | 3.92 |
| 10746 | 1 | 35 | 3 | 4 | 1 | 1 | 0 | 0.2 | 0 | 2.83 | 4.08 | 3.25 | 3.50 | 3.92 |
| 10752 | 1 | 36 | 4 | 6 | 1 | 0 | 200 | 0.3 | 0.5 | 3.58 | 3.17 | 3.83 | 3.67 | 3.75 |
| 10754 | 0 | 24 | 4 | 2 | 1 | 1 | 0 | 0 | 0 | 4.42 | 1.08 | 3.08 | 2.33 | 2.33 |
| 10755 | 0 | 24 | 4 | 4 | 1 | 0 | 600 | 0.2 | 0 | 4.17 | 3.75 | 2.58 | 2.25 | 2.92 |
| 10757 | 0 | 51 | 3 | 4 | 1 | 1 | 0 | 0 | 0 | 2.33 | 3.42 | 3.50 | 4.00 | 3.08 |
| 10758 | 1 | 27 | 3 | 3 | 0 | 2 | 500 | 0.36 | 1 | 2.83 | 3.08 | 3.92 | 3.75 | 3.08 |
| 10762 | 0 | 42 | 4 | 2 | 0 | 0 | 600 | 0.42 | 1 | 2.83 | 3.42 | 3.33 | 4.17 | 3.17 |
| 10764 | 0 | 48 | 3 | 2 | 1 | 0 | 200 | 0.5 | 0.5 | 3.92 | 2.92 | 3.17 | 3.33 | 2.67 |
| 10765 | 0 | 35 | 3 | 2 | 1 | 0 | 500 | 0.5 | 1 | 3.42 | 3.92 | 4.42 | 4.25 | 3.08 |
| 10766 | 0 | 32 | 3 | 2 | 0 | 0 | 400 | 0.2 | 0.5 | 4.75 | 2.08 | 4.17 | 2.58 | 1.92 |
| 10769 | 1 | 44 | 3 | 5 | 0 | 0 | 400 | 0.34 | 0.5 | 3.42 | 2.33 | 3.42 | 3.92 | 2.83 |
| 10778 | 1 | 36 | 4 | 5 | 1 | 0 | 0 | 0 | 1 | 3.83 | 2.83 | 2.92 | 4.08 | 2.42 |
| 10780 | 1 | 24 | 4 | 2 | 0 | 1 | 0 | 0 | 0.5 | 3.17 | 2.67 | 2.83 | 3.75 | 4.17 |
| 10781 | 0 | 57 | 4 | 2 | 0 | 1 | 0 | 0 | 0 | 2.83 | 2.83 | 3.33 | 3.08 | 2.92 |
| 10787 | 0 | 42 | 3 | 4 | 0 | 2 | 500 | 0.46 | 0.5 | 3.25 | 3.17 | 3.92 | 3.75 | 3.50 |
| 10789 | 0 | 20 | 1 | 2 | 1 | 1 | 300 | 0.2 | 0 | 3.25 | 2.92 | 3.58 | 3.33 | 3.08 |
| 10791 | 0 | 42 | 4 | 2 | 0 | 2 | 200 | 0.44 | 0 | 3.17 | 3.00 | 3.50 | 3.42 | 2.92 |
| 10794 | 1 | 28 | 1 | 1 | 0 | 1 | 500 | 0.5 | 1 | 3.50 | 2.92 | 3.67 | 3.25 | 3.33 |
| 10801 | 1 | 38 | 3 | 6 | 1 | 2 | 1000 | 0.46 | 1 | 2.75 | 2.42 | 3.67 | 3.92 | 2.83 |
| 10802 | 1 | 37 | 3 | 4 | 0 | 0 | 400 | 0.16 | 0 | 3.67 | 3.67 | 3.42 | 3.00 | 4.00 |
| 10803 | 1 | 49 | 3 | 4 | 0 | 1 | 1000 | 0.5 | 0 | 2.75 | 2.75 | 4.17 | 4.00 | 3.08 |
| 10808 | 1 | 46 | 4 | 7 | 1 | 1 | 1000 | 0.5 | 1 | 2.58 | 3.92 | 4.58 | 2.92 | 1.92 |
| 10810 | 0 | 54 | 2 | 3 | 1 | 0 | 600 | 0.5 | 1 | 2.92 | 3.50 | 4.17 | 3.67 | 4.00 |
| 10811 | 0 | 43 | 4 | 2 | 1 | 1 | 0 | 0 | 0 | 3.08 | 3.33 | 2.92 | 3.75 | 3.42 |
| 10812 | 0 | 44 | 3 | 2 | 0 | 1 | 500 | 0.4 | 0.5 | 2.75 | 3.33 | 3.75 | 3.50 | 3.83 |
| 10813 | 1 | 51 | 3 | 3 | 1 | 0 | 1000 | 0.6 | 1 | 3.50 | 3.08 | 3.42 | 4.50 | 2.75 |
| 10814 | 0 | 48 | 3 | 4 | 1 | 1 | 1000 | 0.5 | 1 | 3.33 | 3.33 | 3.83 | 4.00 | 3.58 |
| 10816 | 0 | 22 | 2 | 2 | 0 | 1 | 0 | 0 | 0 | 3.83 | 2.92 | 3.42 | 2.75 | 3.08 |
| 10819 | 1 | 36 | 4 | 4 | 1 | 0 | 0 | 0 | 0 | 4.25 | 2.83 | 4.00 | 2.50 | 3.67 |
| 10821 | 0 | 49 | 3 | 2 | 0 | 1 | 0 | 0 | 0.5 | 3.92 | 3.92 | 3.58 | 3.33 | 4.42 |
| 10824 | 1 | 39 | 3 | 5 | 1 | 0 | 1000 | 0.4 | 0.5 | 1.92 | 3.58 | 4.17 | 4.08 | 4.00 |
| 10827 | 1 | 49 | 5 | 2 | 1 | 0 | 1000 | 0.32 | 0.5 | 2.83 | 2.58 | 3.58 | 2.83 | 2.92 |
| 10829 | 1 | 23 | 3 | 4 | 0 | 1 | 0 | 0 | 0 | 3.75 | 2.92 | 4.00 | 2.83 | 3.75 |
| 10832 | 1 | 34 | 3 | 4 | 1 | . | 500 | 0.44 | 1 | 2.50 | 3.17 | 3.83 | 4.42 | 3.08 |
| 10835 | 1 | 51 | 3 | 5 | 1 | 1 | 600 | 0.4 | 1 | 3.00 | 3.17 | 2.83 | 3.67 | 3.17 |
| 10838 | 1 | 34 | 2 | 3 | 0 | 1 | 600 | 0.42 | 0.5 | 3.00 | 2.58 | 2.67 | 3.25 | 2.58 |
| 10839 | 0 | 46 | 3 | 2 | 1 | 0 | 500 | 0.5 | 1 | 3.75 | 2.67 | 4.17 | 3.92 | 3.75 |
| 10847 | 0 | 51 | 4 | 2 | 1 | 0 | 100 | 0 | 0.5 | 3.17 | 3.00 | 3.67 | 4.00 | 3.00 |
| 10848 | 0 | 43 | 2 | 2 | 0 | 1 | 400 | 0.2 | 0 | 2.67 | 3.83 | 4.08 | 4.08 | 4.00 |
| 10849 | 1 | 57 | 4 | 6 | 1 | 1 | 600 | 0.54 | 1 | 3.92 | 2.42 | 3.75 | 3.33 | 3.25 |
| 10850 | 0 | 32 | 3 | 4 | 1 | 1 | 0 | 0 | 0 | 3.08 | 4.17 | 4.17 | 3.67 | 4.25 |
| 10852 | 0 | 33 | 2 | 1 | 0 | 1 | 200 | 0.4 | 0 | 3.33 | 3.42 | 2.33 | 3.50 | 3.17 |
| 10854 | 1 | 40 | 2 | 4 | 1 | 0 | 1000 | 0.52 | 0.5 | 3.33 | 2.17 | 3.50 | 3.58 | 3.25 |
| 10860 | 1 | 39 | 4 | 6 | 1 | 1 | 200 | 0 | 0 | 2.75 | 2.83 | 3.58 | 2.58 | 3.75 |
| 10863 | 0 | 50 | 3 | 2 | 1 | 1 | 1000 | 0.5 | 0.5 | 3.25 | 2.50 | 3.67 | 3.36 | 3.00 |
| 10865 | 1 | 37 | 3 | 5 | 0 | . | 500 | 0.44 | 1 | 3.17 | 2.75 | 3.92 | 3.75 | 3.42 |
| 10867 | 1 | 36 | 2 | 6 | 0 | 2 | 500 | 0.5 | 0.5 | 3.25 | 2.25 | 3.75 | 2.50 | 2.83 |
| 10868 | 0 | 22 | 4 | 3 | 1 | 1 | 500 | 0.3 | 0.5 | 3.67 | 2.42 | 3.58 | 4.08 | 3.00 |
| 10877 | 1 | 24 | 3 | 4 | 0 | . | 200 | 0 | 1 | 4.17 | 3.25 | 3.42 | 3.58 | 3.17 |
| 10879 | 0 | 27 | 4 | 2 | 0 | 1 | 300 | 0 | 0 | 4.33 | 3.25 | 4.33 | 4.33 | 3.83 |
| 10880 | 0 | 53 | 3 | 2 | 1 | 0 | 0 | 0 | 0 | 3.50 | 2.50 | 3.17 | 3.33 | 2.83 |
| 10885 | 1 | 42 | 3 | 4 | 1 | 1 | 0 | 0 | 0.5 | 3.17 | 3.25 | 3.17 | 3.75 | 3.75 |
| 10886 | 1 | 31 | 4 | 6 | 1 | 1 | 1000 | 0.18 | 0.5 | 4.00 | 3.00 | 3.25 | 3.58 | 3.75 |
| 10887 | 0 | 49 | 3 | 1 | 0 | 1 | 400 | 0.38 | 1 | 2.67 | 3.50 | 3.42 | 3.83 | 3.50 |
| 10888 | 0 | 38 | 3 | 3 | 0 | 1 | 700 | 0.5 | 0.5 | 4.25 | 2.83 | 3.33 | 3.58 | 2.92 |
| 10898 | 1 | 29 | 2 | 4 | 1 | 0 | 1000 | 0.48 | 0.5 | 3.58 | 2.83 | 3.50 | 3.08 | 2.75 |
| 20932 | 1 | 55 | 3 | 2 | 1 | 1 | 500 | 0.38 | 0.5 | 3.17 | 3.25 | 3.75 | 4.00 | 3.08 |
| 20936 | 0 | 37 | 4 | 5 | 0 | 1 | 300 | 0.3 | 0 | 2.67 | 3.67 | 4.08 | 3.58 | 3.25 |
| 20944 | 1 | 57 | 4 | 4 | 0 | 1 | 300 | 0.42 | 1 | 3.33 | 3.33 | 3.83 | 3.83 | 3.58 |
| 20948 | 1 | 53 | 4 | 6 | 1 | 0 | 0 | 0 | 1 | 2.50 | 3.33 | 3.33 | 3.83 | 3.92 |
| 20950 | 0 | 50 | 3 | 3 | 1 | 0 | 1000 | 0.46 | 0 | 4.17 | 2.08 | 3.83 | 3.92 | 3.33 |
| 20954 | 0 | 59 | 3 | 2 | 1 | 1 | 500 | 0.5 | 1 | 3.75 | 3.25 | 3.50 | 3.25 | 3.25 |
| 20969 | 1 | 20 | 4 | 2 | 1 | 0 | 1000 | 0.4 | 0.5 | 3.58 | 3.08 | 3.92 | 3.25 | 2.92 |
| 20971 | 0 | 49 | 3 | 2 | 1 | 1 | 1000 | 0.48 | 1 | 3.17 | 2.08 | 3.42 | 4.17 | 3.17 |
| 20972 | 0 | 22 | 3 | 2 | 0 | 1 | 200 | 0.08 | 0.5 | 4.00 | 3.33 | 4.42 | 4.50 | 3.58 |
| 20981 | 1 | 44 | 3 | 4 | 0 | 0 | 200 | 0.42 | 0.5 | 2.67 | 3.42 | 3.33 | 3.50 | 3.75 |
| 20982 | 1 | 27 | 3 | 4 | 1 | 1 | 0 | 0 | 0 | 3.83 | 2.33 | 3.08 | 2.67 | 3.25 |
| 20992 | 1 | 32 | 3 | 4 | 1 | 1 | 100 | 0.1 | 0.5 | 3.17 | 2.17 | 2.83 | 3.50 | 3.00 |
| 20993 | 0 | 22 | 4 | 3 | 1 | 0 | 0 | 0 | 0.5 | 2.58 | 4.25 | 4.25 | 4.67 | 3.58 |
| 20997 | 0 | 24 | 3 | 3 | 1 | 2 | 0 | 0 | 0 | 4.00 | 3.58 | 3.08 | 3.08 | 4.42 |
| 21001 | 1 | 53 | 3 | 6 | 1 | 1 | 500 | 0.4 | 0.5 | 2.83 | 3.08 | 3.67 | 3.67 | 3.50 |
| 21013 | 1 | 34 | 4 | 5 | 0 | 0 | 300 | 0.3 | 1 | 2.25 | 3.67 | 3.08 | 3.67 | 3.83 |
| 21033 | 0 | 24 | 3 | 2 | 1 | 0 | 500 | 0.4 | 1 | 2.75 | 3.25 | 3.50 | 3.58 | 3.25 |
| 21035 | 1 | 46 | 2 | 4 | 0 | 0 | 0 | 0 | 0 | 2.67 | 3.50 | 3.33 | 2.83 | 3.92 |
| 21044 | 1 | 42 | 2 | 4 | 1 | 1 | 100 | 0.2 | 0 | 2.83 | 3.67 | 4.75 | 3.42 | 3.33 |
| 21049 | 0 | 53 | 2 | 4 | 0 | 1 | 400 | 0.4 | 1 | 3.50 | 3.33 | 3.25 | 3.50 | 3.67 |
| 21050 | 1 | 35 | 4 | 5 | 1 | 1 | 1000 | 0.5 | 0 | 3.00 | 3.00 | 2.92 | 3.83 | 4.17 |
| 21055 | 1 | 56 | 3 | 5 | 1 | 2 | 500 | 0.5 | 1 | 3.25 | 2.58 | 3.33 | 3.75 | 3.33 |
| 21057 | 1 | 53 | 4 | 7 | 1 | 1 | 200 | 0.1 | 0.5 | 3.00 | 2.67 | 3.00 | 3.33 | 3.58 |
| 21062 | 0 | 51 | 4 | 2 | 1 | . | 500 | 0.36 | 1 | 2.00 | 4.00 | 3.75 | 4.92 | 4.17 |
| 21068 | 1 | 37 | 2 | 3 | 1 | 0 | 0 | 0.02 | 0.5 | 4.00 | 2.92 | 4.33 | 3.00 | 2.58 |
| 21070 | 1 | 44 | 3 | 5 | 0 | 1 | 0 | 0 | 0 | 3.33 | 2.42 | 3.00 | 2.67 | 3.50 |
| 21076 | 1 | 46 | 2 | 4 | 0 | 0 | 600 | 0.36 | 0 | 4.00 | 2.42 | 3.58 | 3.25 | 3.00 |
| 21077 | 0 | 52 | 3 | 2 | 0 | 1 | 500 | 0.5 | 1 | 3.67 | 3.08 | 4.00 | 4.00 | 3.58 |
| 21080 | 1 | 41 | 2 | 2 | 0 | . | 500 | 0.5 | 0 | 2.58 | 2.00 | 4.00 | 2.58 | 3.42 |
| 21086 | 1 | 30 | 3 | 3 | 1 | 0 | 0 | 0 | 1 | 2.75 | 2.83 | 3.50 | 3.75 | 3.08 |
| 21087 | 0 | 43 | 2 | 3 | 0 | 0 | 300 | 0.4 | 1 | 3.58 | 3.08 | 3.42 | 4.33 | 2.83 |
| 21090 | 0 | 58 | 4 | 4 | 1 | 2 | 200 | 0.5 | 0.5 | 3.38 | 3.00 | 2.43 | 3.00 | 2.86 |
| 21098 | 0 | 30 | 2 | 3 | 0 | 0 | 500 | 0.22 | 0.5 | 3.50 | 2.83 | 3.58 | 4.00 | 3.58 |
| 21100 | 0 | 25 | 2 | 3 | 1 | 1 | 1000 | 0.46 | 1 | 3.92 | 2.50 | 3.17 | 3.25 | 3.08 |
| 21106 | 1 | 28 | 4 | 4 | 1 | 1 | 1000 | 0.56 | 0.5 | 2.92 | 3.75 | 3.92 | 4.25 | 3.00 |
| 21109 | 1 | 38 | 3 | 5 | 1 | 1 | 0 | 0 | 0 | 2.25 | 3.17 | 3.67 | 3.17 | 3.67 |
| 21119 | 1 | 28 | 3 | 5 | 1 | 0 | 700 | 0.44 | 0.5 | 3.50 | 3.08 | 3.75 | 3.67 | 3.42 |
| 21123 | 1 | 35 | 3 | 4 | 1 | 0 | 300 | 0.4 | 0 | 3.75 | 3.58 | 4.17 | 3.75 | 3.08 |
| 21125 | 1 | 54 | 4 | 6 | 1 | 2 | 1000 | 0.4 | 1 | 4.25 | 4.17 | 3.83 | 3.67 | 2.58 |
| 21126 | 0 | 23 | 2 | 3 | 0 | 1 | 800 | 0.48 | 0 | 4.25 | 2.25 | 3.42 | 2.17 | 3.25 |
| 21129 | 1 | 36 | 3 | 4 | 0 | 1 | 0 | 0.08 | 0.5 | 2.58 | 2.42 | 3.00 | 3.25 | 3.33 |
| 21135 | 1 | 49 | 5 | 6 | 1 | . | 1000 | 1 | 1 | 3.92 | 2.83 | 3.17 | 3.50 | 2.50 |
| 21150 | 1 | 43 | 2 | 3 | 1 | 2 | 1000 | 0.5 | 1 | 3.58 | 3.00 | 3.75 | 3.42 | 3.25 |
| 21159 | 1 | 45 | 3 | 7 | 1 | 0 | 600 | 0.4 | 0.5 | 3.75 | 3.33 | 3.08 | 3.17 | 3.42 |
| 21161 | 1 | 31 | 2 | 4 | 1 | 0 | 400 | 0.12 | 0.5 | 3.42 | 3.42 | 3.92 | 4.00 | 3.92 |
| 21167 | 1 | 46 | 2 | 4 | 0 | 1 | 600 | 0.44 | 0 | 2.67 | 3.50 | 4.17 | 3.25 | 3.92 |
| 21190 | 1 | 35 | 3 | 2 | 1 | 1 | 600 | 0.38 | 0.5 | 3.08 | 3.00 | 4.08 | 3.75 | 3.50 |
| 21202 | 1 | 46 | 3 | 5 | 1 | 1 | 0 | 0 | 1 | 2.00 | 3.92 | 4.42 | 4.25 | 3.00 |
| 21207 | 1 | 45 | 2 | 4 | 0 | 0 | 1000 | 0.5 | 1 | 3.50 | 2.42 | 3.58 | 3.33 | 3.00 |
| 21218 | 1 | 31 | 3 | 3 | 1 | 1 | 700 | 0.48 | 1 | 2.83 | 3.75 | 4.08 | 4.08 | 3.25 |
| 21230 | 1 | 52 | 4 | 5 | 1 | 0 | 1000 | 0.4 | 0.5 | 3.58 | 2.50 | 3.58 | 3.50 | 2.33 |
| 21238 | 1 | 29 | 2 | 4 | 1 | 0 | 1000 | 1 | 0.5 | 3.33 | 3.33 | 4.17 | 3.67 | 3.67 |
| 21249 | 1 | 21 | 4 | 1 | 1 | 0 | 0 | 0 | 1 | 3.08 | 3.25 | 3.25 | 3.17 | 2.67 |
| 21257 | 1 | 45 | 4 | 6 | 1 | 1 | 200 | 0.2 | 0 | 3.08 | 3.08 | 3.58 | 3.42 | 3.75 |
| 21260 | 1 | 44 | 2 | 2 | 0 | 1 | 0 | 0 | 1 | 2.75 | 3.33 | 4.08 | 4.00 | 4.17 |
| 21263 | 0 | 22 | 3 | 3 | 1 | 1 | 0 | 0 | 0.5 | 1.83 | 4.42 | 4.17 | 4.50 | 4.33 |
| 21269 | 1 | 56 | 1 | 3 | 1 | 0 | 500 | 0.5 | 0 | 3.33 | 2.33 | 2.67 | 3.17 | 2.58 |
| 21270 | 1 | 39 | 3 | 3 | 1 | 0 | 0 | 0.48 | 0.5 | 2.75 | 2.58 | 3.83 | 3.58 | 3.42 |
| 21273 | 1 | 31 | 2 | 2 | 1 | 0 | 1000 | 0.5 | 1 | 3.83 | 2.58 | 4.83 | 3.75 | 3.25 |
| 21274 | 1 | 42 | 4 | 6 | 1 | 0 | 300 | 0.32 | 0 | 3.08 | 3.83 | 3.50 | 3.33 | 3.33 |
| 21289 | 1 | 58 | 3 | 4 | 1 | 0 | 500 | 0.1 | 1 | 2.83 | 3.42 | 3.42 | 3.50 | 3.58 |
| 21297 | 1 | 27 | 3 | 4 | 1 | 1 | 0 | 0 | 1 | 3.42 | 2.92 | 3.67 | 3.08 | 2.42 |
| 21298 | 0 | 29 | 1 | 3 | 0 | 1 | 200 | 0 | 0 | 3.75 | 3.33 | 3.17 | 3.50 | 3.33 |
| 21305 | 1 | 49 | 3 | 3 | 0 | 0 | 500 | 0.5 | 1 | 3.50 | 3.00 | 3.83 | 3.67 | 2.92 |
| 21306 | 0 | 42 | 3 | 2 | 0 | 1 | 500 | 0.5 | 0.5 | 1.83 | 4.33 | 2.58 | 4.17 | 3.67 |
| 21312 | 1 | 31 | 3 | 4 | 1 | 1 | 0 | 0.4 | 0.5 | 4.00 | 3.17 | 3.92 | 3.83 | 3.42 |
| 21318 | 1 | 53 | 3 | 3 | 1 | 0 | 0 | 0 | 1 | 3.17 | 3.33 | 2.92 | 4.50 | 4.00 |
| 21323 | 0 | 52 | 4 | 2 | 1 | 2 | 100 | 0.5 | 0 | 3.50 | 3.42 | 4.33 | 3.58 | 3.50 |
| 21324 | 1 | 39 | 3 | 6 | 1 | 0 | 400 | 0.5 | 0.5 | 2.83 | 4.50 | 3.42 | 4.08 | 3.58 |
| 21325 | 0 | 53 | 2 | 3 | 0 | 1 | 600 | 0.5 | 0.5 | 3.00 | 2.83 | 2.83 | 3.33 | 3.58 |
| 21328 | 1 | 26 | 3 | 4 | 0 | 0 | 0 | 0.16 | 0 | 2.67 | 3.67 | 3.67 | 2.25 | 3.33 |
| 21331 | 1 | 27 | 3 | 4 | 1 | 1 | 1000 | 0.4 | 0 | 3.83 | 3.08 | 4.08 | 3.92 | 2.75 |
| 21343 | 0 | 43 | 2 | 3 | 1 | 0 | 700 | 0.4 | 1 | 3.42 | 3.42 | 4.17 | 3.75 | 3.08 |
| 21351 | 1 | 28 | 4 | 5 | 1 | 1 | 0 | 0 | 1 | 3.92 | 2.83 | 2.67 | 3.50 | 3.75 |
| 21352 | 0 | 28 | 2 | 3 | 1 | 1 | 0 | 0 | 0 | 3.75 | 3.08 | 3.50 | 3.00 | 3.17 |
| 21360 | 1 | 46 | 3 | 6 | 1 | . | 1000 | 0.46 | 0 | 4.00 | 3.00 | 4.50 | 3.50 | 2.00 |
| 21368 | 1 | 47 | 3 | 4 | 1 | 0 | 500 | 0.5 | 0.5 | 3.42 | 2.58 | 4.00 | 2.50 | 3.58 |
| 21377 | 0 | 53 | 3 | 3 | 0 | 1 | 500 | 0.32 | 0.5 | 2.75 | 3.75 | 3.00 | 4.17 | 3.83 |
| 21381 | 1 | 47 | 4 | 5 | 0 | 0 | 600 | 0.4 | 0 | 3.00 | 2.50 | 3.33 | 3.58 | 3.33 |
| 21391 | 1 | 52 | 3 | 5 | 1 | 1 | 0 | 0.5 | 0 | 2.58 | 3.42 | 3.67 | 4.42 | 3.42 |
| 21394 | 0 | 33 | 4 | 5 | 1 | 0 | 0 | 0 | 1 | 2.42 | 3.75 | 3.75 | 3.92 | 4.08 |
| 21402 | 1 | 52 | 3 | 5 | 1 | 0 | 500 | 0.5 | 0 | 2.17 | 4.00 | 3.42 | 3.67 | 3.67 |
| 21404 | 1 | 55 | 4 | 6 | 1 | 1 | 300 | 0.3 | 0.5 | 2.25 | 3.58 | 3.67 | 3.67 | 3.25 |
| 21413 | 0 | 26 | 1 | 2 | 0 | 0 | 1000 | 0.5 | 0.5 | 4.42 | 2.33 | 4.42 | 3.58 | 2.67 |
| 21415 | 1 | 41 | 2 | 4 | 0 | 1 | 300 | 0.04 | 0 | 3.33 | 3.00 | 3.42 | 3.25 | 3.25 |
| 21416 | 1 | 53 | 3 | 6 | 1 | 1 | 500 | 0.3 | 1 | 2.25 | 3.75 | 3.25 | 3.00 | 3.67 |
| 21418 | 1 | 36 | 3 | 4 | 1 | 2 | 500 | 0.46 | 0.5 | 3.58 | 3.25 | 3.50 | 3.75 | 3.50 |
| 21420 | 1 | 51 | 3 | 4 | 0 | 1 | 700 | 0.5 | 0 | 3.75 | 3.17 | 3.50 | 3.42 | 3.75 |
| 21422 | 0 | 31 | 4 | 3 | 1 | 1 | 500 | 0.46 | 0 | 3.92 | 1.67 | 4.58 | 3.17 | 2.58 |
| 21423 | 1 | 52 | 3 | 7 | 1 | 1 | 500 | 0.38 | 0 | 3.17 | 2.50 | 4.00 | 2.75 | 2.58 |
| 21424 | 0 | 23 | 3 | 2 | 1 | 0 | 300 | 0 | 0.5 | 2.83 | 2.25 | 3.50 | 3.25 | 2.58 |
| 21430 | 1 | 26 | 3 | 3 | 1 | . | 0 | 0 | 1 | 3.17 | 2.75 | 4.25 | 2.83 | 3.50 |
| 21433 | 1 | 39 | 3 | 5 | 1 | 0 | 500 | 0 | 0 | 2.75 | 3.25 | 3.75 | 3.25 | 3.33 |
| 21444 | 1 | 29 | 3 | 4 | 1 | 0 | 200 | 0.22 | 0 | 3.67 | 2.25 | 3.50 | 3.42 | 1.92 |
| 21447 | 1 | 27 | 4 | 4 | 1 | 0 | 0 | 0 | 1 | 1.83 | 4.67 | 3.83 | 4.67 | 4.33 |
| 21455 | 1 | 23 | 2 | 4 | 0 | 1 | 0 | 0.28 | 0 | 4.33 | 2.33 | 3.17 | 2.92 | 3.08 |
| 21459 | 1 | 53 | 4 | 6 | 1 | 0 | 500 | 0.5 | 1 | 3.08 | 3.83 | 4.08 | 3.83 | 3.92 |
| 21462 | 1 | 51 | 4 | 7 | 1 | 1 | 300 | 0.24 | 1 | 2.50 | 3.92 | 4.17 | 4.00 | 3.33 |
| 21464 | 1 | 36 | 3 | 4 | 0 | 0 | 200 | 0.5 | 0 | 3.92 | 2.17 | 2.92 | 4.00 | 3.75 |
| 21472 | 0 | 52 | 3 | 5 | 1 | 0 | 1000 | 0.44 | 1 | 2.33 | 3.75 | 4.08 | 4.33 | 3.17 |
| 21473 | 1 | 29 | 2 | 2 | 1 | 0 | 0 | 0 | 0 | 4.42 | 2.33 | 4.17 | 4.08 | 2.17 |
| 21476 | 1 | 55 | 4 | 5 | 0 | 1 | 1000 | 0.42 | 1 | 3.17 | 3.58 | 4.17 | 3.92 | 3.83 |
| 21479 | 1 | 50 | 2 | 1 | 0 | . | 100 | 0.26 | 0 | 2.92 | 3.50 | 4.08 | 3.33 | 3.58 |
| 21481 | 0 | 31 | 4 | 4 | 0 | 1 | 200 | 0.34 | 1 | 4.00 | 2.25 | 3.67 | 2.92 | 2.75 |
| 21495 | 1 | 32 | 1 | 3 | 1 | 2 | 1000 | 0 | 1 | 3.33 | 3.58 | 3.83 | 3.50 | 3.42 |
| 21498 | 0 | 43 | 4 | 5 | 1 | 1 | 1000 | 0.46 | 1 | 2.83 | 3.75 | 3.83 | 3.92 | 3.42 |
| 21500 | 1 | 44 | 3 | 5 | 1 | 1 | 800 | 0.94 | 1 | 1.92 | 3.50 | 3.67 | 3.83 | 4.00 |
| 21504 | 0 | 23 | 3 | 3 | 0 | 1 | 500 | 0.5 | 0 | 3.67 | 3.08 | 3.67 | 3.25 | 3.58 |
| 21505 | 1 | 32 | 2 | 4 | 1 | 0 | 1000 | 0.4 | 0.5 | 4.33 | 2.17 | 3.50 | 3.33 | 1.67 |
| 21507 | 1 | 52 | 4 | 6 | 1 | 0 | 0 | 0 | 0.5 | 3.33 | 3.08 | 3.17 | 3.08 | 3.67 |
| 21516 | 1 | 43 | 1 | 2 | 0 | 1 | 0 | 0.4 | 0 | 4.08 | 1.33 | 3.75 | 2.42 | 2.67 |
| 21520 | 0 | 25 | 2 | 3 | 1 | . | 1000 | 0.1 | 0 | 4.50 | 2.75 | 4.00 | 2.83 | 2.50 |
| 21533 | 1 | 34 | 3 | 2 | 1 | 0 | 400 | 0.44 | 0 | 3.17 | 2.33 | 4.50 | 3.50 | 3.33 |
| 21535 | 1 | 49 | 4 | 7 | 1 | 2 | 0 | 0 | 0 | 2.58 | 3.25 | 3.42 | 4.08 | 3.83 |
| 21538 | 0 | 36 | 3 | 3 | 1 | 1 | 500 | 0.5 | 1 | 4.42 | 3.58 | 4.17 | 3.75 | 3.75 |
| 21542 | 1 | 50 | 3 | 6 | 1 | 0 | 1000 | 0.5 | 0 | 3.29 | 3.86 | 3.71 | 3.43 | 3.71 |
| 21546 | 0 | 50 | 3 | 2 | 1 | 1 | 500 | 0.4 | 0.5 | 3.50 | 2.42 | 3.92 | 3.92 | 2.58 |
| 21549 | 0 | 29 | 3 | 4 | 1 | 0 | 500 | 0.58 | 0.5 | 4.00 | 2.42 | 4.50 | 3.25 | 3.58 |
| 21552 | 0 | 36 | 3 | 4 | 1 | 2 | 400 | 0.42 | 1 | 3.83 | 3.33 | 2.92 | 3.42 | 3.25 |
| 21554 | 1 | 22 | 1 | 2 | 1 | 1 | 500 | 0.26 | 1 | 2.83 | 3.42 | 3.58 | 3.83 | 3.00 |
| 21555 | 1 | 32 | 2 | 3 | 1 | 0 | 200 | 0 | 0 | 3.08 | 2.42 | 3.92 | 3.17 | 3.83 |
| 21556 | 1 | 21 | 3 | 2 | 1 | . | 100 | 0.02 | 0 | 3.25 | 3.25 | 3.67 | 4.25 | 2.75 |
| 21571 | 1 | 54 | 3 | 5 | 1 | 0 | 300 | 0.38 | 0 | 3.92 | 1.50 | 3.92 | 3.08 | 3.33 |
| 21577 | 1 | 50 | 4 | 6 | 1 | 0 | 700 | 0.46 | 0 | 3.75 | 4.00 | 4.25 | 3.00 | 2.42 |
| 21578 | 0 | 22 | 3 | 4 | 0 | 0 | 200 | 0.02 | 0 | 4.08 | 3.58 | 3.33 | 2.67 | 3.50 |
| 21588 | 0 | 26 | 3 | 4 | 1 | . | 500 | 0.54 | 1 | 3.17 | 3.83 | 3.42 | 3.92 | 2.92 |
| 21594 | 1 | 45 | 3 | 5 | 1 | 2 | 100 | 0 | 0 | 3.58 | 2.83 | 2.58 | 3.50 | 2.83 |
| 21605 | 1 | 24 | 2 | 3 | 1 | 2 | 0 | 0.02 | 0 | 4.67 | 2.42 | 4.42 | 2.83 | 3.58 |
| 21606 | 1 | 50 | 3 | 5 | 0 | 2 | 1000 | 0.34 | 1 | 2.50 | 3.75 | 4.17 | 4.08 | 3.50 |
| 21608 | 1 | 28 | 3 | 4 | 1 | 1 | 0 | 0.5 | 0 | 3.33 | 3.25 | 4.17 | 2.92 | 3.50 |
| 21617 | 0 | 45 | 2 | 2 | 0 | 0 | 700 | 0 | 1 | 3.58 | 2.92 | 3.75 | 3.58 | 3.17 |
| 21619 | 1 | 46 | 4 | 6 | 0 | 0 | 500 | 0.26 | 0.5 | 3.33 | 3.42 | 3.08 | 3.67 | 4.00 |
| 21634 | 1 | 48 | 3 | 5 | 1 | 0 | 500 | 0.5 | 1 | 4.25 | 3.83 | 4.58 | 3.83 | 4.17 |
| 21637 | 0 | 49 | 3 | 5 | 1 | 0 | 200 | 0 | 0.5 | 3.25 | 3.33 | 3.33 | 3.08 | 2.92 |
| 21640 | 0 | 29 | 2 | 3 | 1 | 1 | 500 | 0.34 | 0.5 | 3.33 | 3.33 | 4.33 | 3.75 | 2.92 |
| 21654 | 0 | 28 | 2 | 3 | 1 | 0 | 500 | 0.2 | 0 | 3.67 | 2.92 | 4.50 | 3.58 | 3.92 |
| 21661 | 1 | 46 | 3 | 5 | 1 | 0 | 100 | 0.1 | 0 | 4.00 | 1.92 | 3.83 | 2.67 | 3.75 |
| 21667 | 0 | 57 | 2 | 3 | 0 | 0 | 500 | 1 | 0 | 3.58 | 2.67 | 3.33 | 3.08 | 3.42 |
| 21671 | 1 | 36 | 2 | 2 | 1 | 1 | 500 | 0.12 | 0 | 4.42 | 2.75 | 3.25 | 2.50 | 2.67 |
| 21688 | 1 | 44 | 3 | 4 | 1 | 0 | 900 | 0.9 | 0 | 3.25 | 3.50 | 3.50 | 3.33 | 3.25 |
| 21690 | 0 | 55 | 4 | 5 | 1 | 1 | 200 | 0.38 | 0.5 | 2.58 | 4.33 | 4.00 | 4.25 | 3.75 |
| 21700 | 1 | 42 | 3 | 5 | 1 | 0 | 200 | 0 | 1 | 2.75 | 2.58 | 3.92 | 4.08 | 3.25 |
| 21729 | 1 | 36 | 1 | 3 | 1 | 1 | 0 | 0 | 0 | 3.92 | 2.75 | 4.25 | 3.83 | 3.50 |
| 21734 | 1 | 41 | 3 | 6 | 1 | 0 | 600 | 0.5 | 1 | 2.83 | 3.42 | 3.75 | 4.17 | 3.42 |
| 21754 | 1 | 36 | 3 | 6 | 1 | 0 | 500 | 0.44 | 0.5 | 2.50 | 2.42 | 3.67 | 3.50 | 3.33 |
| 21762 | 1 | 40 | 4 | 4 | 0 | 1 | 900 | 0.44 | 1 | 2.17 | 3.08 | 3.67 | 3.92 | 3.17 |
